# HLA-C–derived peptide MH-1 as an early-stage intervention against SARS-CoV-2 infection

**DOI:** 10.1186/s10020-026-01434-3

**Published:** 2026-02-16

**Authors:** Ting-Yan Jian, Sheng-Yu Huang, Chia-Yu Chang, Chih-Heng Huang, Lik Voon Kiew, Yu-Ling Lin, Yueh-Te Lin, Yen-Chin Liu, Yen-Chen Chen, Yi-Xuan Huang, Hao-Syun Chou, Sook Fan Yap, An-Yu Chen, Yen-Chen Chen, Yu-Chuan Liang, Yu-An Kung, Pei-Yu Wang, Peng-Nien Huang, Chung-Guei Huang, Chia-Ching Chang, Shin-Ru Shih

**Affiliations:** 1 Agricultural Biotechnology Research Center, Academia Sinica, 128 Academia Road, Section 2, Nankang Dist, Taipei, 11529 Taiwan; 2 Research Center for Emerging Viral Infections, College of Medicine, Chang Gung University, Taoyuan, 333323 Taiwan; 3 Department of Medical Biotechnology and Laboratory Science, College of Medicine, Chang Gung University, Taoyuan, 333323 Taiwan; 4 Department of Biological Science and Technology, National Yang Ming Chiao Tung University, BioICT building room 537, No. 75, Bo-ai Street, East Dist, Hsinchu, 30068 Taiwan; 5 Center for Intelligent Drug Systems and Smart Bio-devices (IDS2B), National Yang Ming Chiao Tung University, Hsinchu, 30068 Taiwan; 6 Institute of Preventive Medicine, National Defense Medical University, Taipei, 237010 Taiwan; 7 Graduate Institute of Medical Sciences, National Defense Medical University, Taipei, 114201 Taiwan; 8 Department of Microbiology and Immunology, National Defense Medical University, Taipei, 114201 Taiwan; 9 Department of Pharmacology, Faculty of Medicine, University of Malaya, Kuala Lumpur, 50603 Malaysia; 10 Centre of Excellence for Innovative Medical Devices, University of Malaya, Kuala Lumpur, 50603 Malaysia; 11 Department of Preclinical Sciences, M. Kandiah Faculty of Medicine and Health Sciences, University of Tunku Abdul Rahman, Kajang, Selangor 43000 Malaysia; 12 WLT-Centre for Research in Communicable Diseases, University of Tunku Abdul Rahman, Kajang, Selangor 43000 Malaysia; 13 Division of Infectious Diseases, Department of Pediatrics, Linkou Chang Gung Memorial Hospital, Taoyuan, 333423 Taiwan; 14 International Master Degree Program for Molecular Medicine in Emerging Viral Infections, Chang Gung University, Taoyuan, 333323 Taiwan; 15 Department of Laboratory Medicine, Linkou Chang Gung Memorial Hospital, Taoyuan, 333423 Taiwan; 16 Department of Electrophysics, National Yang Ming Chiao Tung University, Hsinchu, 30010 Taiwan; 17 International College of Semiconductor Technology, National Yang Ming Chiao Tung University, Hsinchu, 30010 Taiwan; 18 Institute of Physics, Academia Sinica, Taipei, 10529 Taiwan; 19 Institute of Biomedical Sciences and Emerging Infectious Diseases Division (EIDD), Biomedical Translation Research Center (BioTReC), AS, Taipei, 11571 Taiwan; 20 Graduate Institute of Biomedical Science, College of Medicine, Chang Gung University, Taoyuan, Taiwan; 21 Science/Graduate Program of Biomedical Science/Research Center for Emerging Viral Infections, Chang Gung University, 259 Wen-Hwa 1st Road, Kwei-Shan Dist, Tao-Yuan City, 33302 Taiwan; 22 Emerging Infectious Disease Division (EIDD), Biomedical Translation Research Center (BioTReC), Academia Sinica, No. 99, Ln. 130, Sec. 1, Academia Rd., Nangang Dist, Taipei, 11529 Taiwan

**Keywords:** HLA-C, SARS-CoV-2, T-lymphocytes, hACE2-mice, S-RBD

## Abstract

**Background:**

Emerging evidence suggests that preventing SARS-CoV-2 from entering and infecting host cells represents an effective strategy to limit viral infection, particularly in the context of its ongoing evolution. In this study, a small peptide fragment derived from major histocompatibility complex class I (MHC class I), designated MH-1, was investigated for its ability to interfere with the early stages of SARS-CoV-2 infection.

**Methods:**

Molecular docking was used to characterize the interaction between MH-1 and the receptor-binding domain (RBD) of the SARS-CoV-2 spike (S) protein. The inhibitory effect of MH-1 on S protein–ACE2 binding was further evaluated using an ACE2-functionalized electrochemical impedance spectroscopy (EIS) biosensing platform. Antiviral efficacy was assessed using SARS-CoV-2 S-pseudotyped lentiviruses and SARS-CoV-2 variants in different human cells. In vivo inhibitory efficacy of MH-1 was assessed in the K18-hACE2 mouse model, followed by lung viral load measurement and histopathological assessment.

**Results:**

MH-1 peptide interacted with the S-RBD and disrupted S protein-ACE2 binding. MH-1 effectively reduced SARS-CoV-2 infection in cells that expressed different levels of ACE2 and TMPRSS2. Furthermore, MH-1 decreased the infection of SARS-CoV-2 in T lymphocytes that highly express HLA-C but have low levels of ACE2 and TMPRSS2. In animal studies, MH-1 reduced the viral load in the lungs of K18-hACE2 mice and reduced the infiltration of immune cells, including macrophages and T cells, into the lungs. Levels of lung damage and inflammatory cytokines were also reduced by MH-1 and restored to normal.

**Conclusions:**

These findings identify MH-1 as a promising prophylactic or early-stage intervention that inhibits SARS-CoV-2 infection by interfering with spike-mediated infection of pulmonary and immune cells.

**Supplementary Information:**

The online version contains supplementary material available at 10.1186/s10020-026-01434-3.

## Introduction

The World Health Organization (WHO) declared that COVID-19 no longer constitutes a Public Health Emergency of International Concern (PHEIC). This reflected a transition from an acute global health crisis to an ongoing and manageable health challenge (Organization 2023). Despite this shift, COVID-19 remains endemic in many regions worldwide and is a major health risk to vulnerable populations, including unvaccinated children, older individuals with weakened immune systems, and immunocompromised persons (Are et al. 2023). Hence, efforts to develop effective therapeutics capable of attenuating COVID-19 severity and complications are ongoing.

Elucidating the SARS-CoV-2 cell entry mechanisms is vital for developing viral cell entry inhibitors, which are a major emerging class of antiviral therapeutics. Several SARS-CoV-2 cell entry mechanisms have been reported (Kung et al. 2022). It is well known that SARS-CoV-2 may bind to the cell surface receptor angiotensin-converting enzyme 2 (ACE2) through its spike (S) protein, thereby triggering the detachment of the S1 subunit from the viral S-protein and exposing the S2 subunit (Hoffmann et al. 2020; Zhou et al. 2020). Viral cell entry directly through the transmembrane protease, serine 2 (TMPRSS2)-mediated cleavage of the S2 subunit, followed by the insertion of the S2 fusion peptide into the target membrane to initiate membrane fusion and viral RNA cytoplasmic release (Takeda 2022). In the absence of TMPRSS2, SARS-CoV-2 indirectly enters the cell through the clathrin-mediated endocytosis of the virus-ACE2 complex (Bayati et al. 2021), followed by S2 cleavage by the endolysosomal cathepsins (Hoffmann et al. 2020) to initiate virus-endolysosome fusion and viral RNA content release into the cytoplasm.

For tissues with low ACE2 or TMPRSS2 levels, SARS-CoV-2 may enter host cells through alternative pathways. SARS-CoV-2 binds to soluble ACE2 (sACE2) or vasopressin in the blood to form virus-ACE2 or virus-ACE2-vasopressin complexes, then interacts with angiotensin II receptor type 1 (AT1) or vasopressin V1b (AVPR1B) receptors to enter cells through receptor-mediated endocytosis (Yeung et al. 2021). Integrin α5β1 is considered a route for SARS-CoV-2 entry due to a similar binding affinity to ACE2 (Liu et al. 2022). SARS-CoV-2 attaches to neuropilin-1 (NRP1) on the surface of olfactory endothelial cells through the polybasic conserved C-terminal regular peptide of the S protein and enters cells through endocytosis, causing neurological SARS-CoV-2 symptoms (Cantuti-Castelvetri et al. 2020; Davies et al. 2020). NRP1 also enhances TMPRSS2-mediated entry of the prototype SARS-CoV-2 through its binding to the multibasic furin-cleavage site of the S1 subunit, which promotes S1 shedding and exposes the S2 subunit to TMPRSS2 (Li and Buck 2021). In addition, the human transferrin receptor (TfR), a ubiquitously distributed molecule, also serves as a receptor for SARS-CoV-2 and facilitates viral entry independently of ACE2 into host cells, thereby providing the virus another mode of infection (Liao et al. 2024).

HLA-C is a classical major histocompatibility complex (MHC) class I molecule that presents endogenous peptides to CD8⁺ T cells and plays a critical role in antiviral immune responses (Parham 2005). In addition to antigen presentation, HLA-C serves as a key ligand for inhibitory and activating killer cell immunoglobulin-like receptors (KIRs) expressed on natural killer (NK) cells, thereby regulating the activation of NK cells and immune surveillance (Long et al. 2013). HLA-C is expressed on nearly all nucleated cells (Wieczorek et al. 2017; Hewitt 2003), and it is abundant in immune cells, particularly lymphocytes. Compared with other classical MHC class I molecules, HLA-C exhibits relatively lower surface expression but has a particularly important function in modulating innate and adaptive immunity (Parham 2005). Evidence suggests that viral pathogens can use or modulate HLA-C-mediated pathways to evade immune recognition (Kulkarni et al. 2011). Notably, SARS-CoV-2 has been shown to actively suppress the expression of MHC class I molecules, thereby weakening antigen presentation and evading cytotoxic immune responses (Moriyama et al. 2023; Schirmeister et al. 2025), highlighting the potential importance of HLA-C-mediated mechanisms in SARS-CoV-2 host-virus interactions.

Despite progress in understanding the mechanisms of SARS-CoV-2 cell entry, further development of effective therapeutics is needed to inhibit viral activity and reduce its ability to infect host cells. Identifying potent inhibitors targeting key protein-protein interactions is an important strategy for antiviral and therapeutic development. However, traditional screening methods are often labor-intensive, time-consuming, and costly, limiting their application in rapid drug discovery. Recent advances in biosensor technology offer attractive alternatives for high-throughput, sensitive detection of molecular interactions. In particular, palladium nanofilm (Pd-NTF) electrodes fabricated on polyethylene terephthalate substrates provide a stable surface-active platform that enables efficient bioconjugation and electrochemical impedance spectroscopy (EIS)-based readout with sub-nanogram sensitivity (Chang et al. 2019). We have developed an ACE2-functionalized Pd-NTF EIS biosensor to monitor the binding of the SARS-CoV-2 S protein to its cellular receptor, ACE2, and to screen potential inhibitory compounds (Kiew et al. 2021). This Pd-NTF EIS biosensor platform can also be used to quickly and efficiently screen inhibitory peptides, providing a supportive tool to accelerate drug development before cell-based functional assays or animal studies.

Our recent search for SARS-CoV-2 cell entry inhibitors among small peptides targeting the S-RBD using phage display techniques (Yang et al. 2021) led to the discovery of an MHC class I (HLA-C) protein-derived small peptide (designated MH-1) that effectively binds to the S-protein, thereby reducing SARS-CoV-2 infectivity in various cell types. MH-1 inhibited infection in mice and reduced lung inflammation. Validation in cell and animal models demonstrates that our developed EIS biosensor can rapidly and sensitively detect the interaction between RBD and MH-1 molecules, enabling the screening of peptides that effectively inhibit early stages of SARS-CoV-2 infection. Furthermore, this study suggests that HLA-C may be a pathway for SARS-CoV-2 to facilitate viral infection in T lymphocytes that highly express HLA-C but lack ACE2 and TMPRSS2 expression.

## Materials and methods

### Molecular docking of S-RBD and MH-1 peptide

Molecular docking helps us predict whether the MH-1 peptide can bind to the S-RBD of SARS-CoV-2. The 3D structure of the S-RBD of SARS-CoV-2 (PDB ID: 8XYZ, chain A) was obtained from the RCSB Protein Data Bank (RCSB PDB). The 3D structure of the MH-1 peptide was modeled using the SWISS-MODEL web tool. The MH-1 peptide was docked with SARS-CoV-2 RBD by HADDOCK2.4 software (https://rascar.science.uu.nl/haddock2.4/) with default parameters, following the standard HADDOCK workflow. Briefly, the docking protocol consists of three stages: (i) rigid-body docking (it0), (ii) semi-flexible refinement (it1), and (iii) final refinement in explicit solvent.

In the rigid-body docking stage, 1,000 initial poses were generated, from which 200 structures with the lowest intermolecular energies were selected for semi-flexible refinement, as implemented in the default HADDOCK2.4 pipeline. The final docked models were clustered based on interface root-mean-square deviation (iRMSD), and clusters were ranked according to the HADDOCK score, which integrates van der Waals, electrostatic, desolvation, and restraint violation energies. The top-ranked cluster with the lowest HADDOCK score and the highest cluster size was selected for further analysis (Honorato et al. 2024). The docking result was visualized using the PyMOL molecular visualization system.

### Recombinant expression and characterization of SARS-CoV-2 S-protein

The S-RBD of SARS-CoV-2 (Wuhan, Delta, and Omicron variants) and ACE2 (the target receptor for S-RBD) were recombinantly expressed in *Escherichia coli* BL21 (DE3) using a T7 RNA polymerase–based expression system (Kiew et al. 2021; Chang et al. 2019). Briefly, the gene fragments for S-RBD and ACE2 were synthesized using a chemical synthesis method (Genomics Co., Taipei, Taiwan) and subcloned into pUC expression vectors (Fig. S1). The resulting vectors were then transformed into XLI-blue-stranded *E. coli* hosts (Chang et al. 2019). The transformed cells were induced with 0.5 mM isopropyl β-D-thiogalactopyranoside (IPTG) in 250 mL Luria-Bertani (LB) medium at 37 °C for 18 h to overexpress the recombinant S-RBD proteins. Recombinant Wuhan, Delta, and Omicron S-RBD proteins, as well as ACE2, were isolated from bacterial inclusion bodies and solubilized in a denaturing buffer. The denatured proteins were then refolded by gradually altering the concentration of the denaturant and salts in the solution and reducing the pH of the solution from 12 to 8.8 (Chang et al. 2002). The refolded proteins were further purified using Ni-NTA affinity chromatography. Briefly, protein samples were incubated with Ni-NTA agarose beads and washed with phosphate buffer (pH 7.5) containing 0.5 M NaCl. Bound proteins were eluted with 0.25 M imidazole in phosphate-buffered saline. To confirm the identity of the refolded Wuhan, Delta, and Omicron S-RBD proteins and ACE2, western blotting was performed using anti-S-protein and anti-ACE2 antibodies (Fig. S2). The MH-1-mcherry was identified by Western blotting with anti-HLAC antibody (Fig. S2). Protein bands were visualized using the NBT/BCIP chromogenic substrate (Sigma‑Aldrich, Cat. No. B1911). Its purity was assessed by SDS-PAGE. Ultraviolet-visible (UV-vis) spectroscopy (λ = 280 nm) was used to estimate the protein concentration.

### Fabrication of an EIS-based biosensor

A Pd-NTF electrode was prepared according to a previously reported method (Kiew et al. 2021). Palladium thin-film electrodes were supplied by Pin Jye Biotech Co., Ltd., Taiwan. The average thickness of the palladium film was 25 nm, and sputtering was performed on the polyethylene (PET) backplane, with the electrode sheet resistance measuring 40 Ω/square. The electrode was then coated with a layer of recombinant ACE2 (serving as a probe layer for S-protein binding through the formation of Pd-S bonds between ACE2 and the active Pd surface) by adding 2 µL of the recombinant ACE2 (0.6 mg/mL) onto the Pd-NTF electrode and incubating at 25 °C for 20 min (Kiew et al. 2021; Chang et al. 2019). Subsequently, the coated electrode was rinsed with double-distilled water and blocked with 1 µL of 1-octadecanethiol (0.1 mM) at 25 °C for 10–15 min. Finally, the ACE2–Pd-NTF electrode was connected to an impedance spectroscopy device/amplifier to complete the ACE2–Pd-NTF biosensor-EIS setup. Successful coating of ACE2 onto Pd-NTF was confirmed by an increase in the R_ct_ of Pd-NTF after ACE2 coating (Chang et al. 2019). The ACE2–Pd-NTF biosensor–EIS setup was validated for interaction selectivity by exposing the biosensing electrode to (i) increasing concentrations of S-RBD protein (Wuhan, Delta, and Omicron variants) to visualize concentration-dependent interactions (Fig. 2B–D) and (ii) increasing concentrations of albumin and lysozyme (as negative controls) to confirm the absence of nonspecific protein binding (Fig. S3).

### Investigation of MH-1 interference on the SARS-CoV-2 S-RBD–ACE2 binding via EIS-based biosensing platform

To determine whether MH-1 in its free form interferes with the binding of SARS-CoV-2 S-RBD to immobilize ACE2, the ACE2-Pd-NTF electrode was pretreated with 2 µL of PBS for 10 min at 25 °C, gently rinsed with distilled water, drained, and connected to the EIS device for baseline impedance measurement. Subsequently, several ACE2-Pd-NTF electrodes were treated with mixtures of MH-1 and S-RBD (Wuhan, Delta, and Omicron BA.2 variants at a concentration of 50 µg/mL) at increasing MH-1 concentrations (0.01–1 mg/mL, 2 µL per electrode) for 10 min at 25 °C, followed by EIS measurement. The net changes in the R_ct_ value ($\:\varDelta\:$R_ct_) were calculated by subtracting the baseline R_ct_ signals of the ACE2–Pd-NTF electrode from those of the electrodes treated with the protein mixtures. A concentration-response curve plotting MH-1 concentration against $\:\varDelta\:$R_ct_ was used to visualize the effect of MH-1 on the binding of S-RBD to ACE2. The interactions between MH-1 and the SARS-CoV-2 S-RBD, as well as between ACE2 and the SARS-CoV-2 S-RBD, were measured using the palladium thin-film electrode to determine the Kd through changes in electrical impedance. Similarly, the inhibitory effect of MH-1 on the binding of S-RBD and ACE2 (IC_50_) was assessed by analyzing impedance changes caused by electrochemical reactions.

### Preparation of SARS-CoV-2-S Luc pseudo-typed lentiviruses

VSV-G, B.1.1.7 (Alpha), BA.1.1.529 (Omicron), BA.4/BA.5 (Omicron) and XBB.1.16 (Omicron) SARS-CoV-2-S Luc pseudo-typed lentiviruses were purchased from the RNA Technology Platform and Gene Manipulation Core (Academia Sinica, Taipei, Taiwan). The vectors pcDNA3.1, pCMVdeltaR8.91, and pLAS2w.FLuc.Ppuro were used to express the S protein on the viral surface. The SARS-CoV-2-S Luc pseudo-typed lentivirus exhibited specific S protein mutations (as listed in Table S1). For the subsequent experiments, lentiviral particles pseudotyped with VSV-G were used as a positive control for viral infection, and lentiviral particles lacking an envelope protein were used as a negative control. SARS-CoV-2 variants are abbreviated as follows: B.1.1.7 (Alpha), Alpha; BA.1.1.529 (Omicron), BA.1; BA.4/BA.5 (Omicron), BA.4/5; and XBB.1.16 (Omicron), XBB.

### SARS-CoV-2 viruses

SARS-CoV-2/human/TWN/CGMH-CGU-01/2020 (Wuhan) (GISAID accession number: EPI_ISL_411915; NCBI accession number: MT192759.1), Alpha (B.1.1.7), and Omicron (BA.1) were originally isolated from patients with COVID-19 at Chang Gung Memorial Hospital. The BA.5 variant (hCoV-19/Taiwan/689423/2022) was obtained from the Taiwan Food and Drug Administration, and XBB. 1.1 (hCoV-19/Taiwan/984581/2022) was obtained from the Taiwan Center for Disease Control (Taiwan CDC). Viral amplification and manipulation were performed in accredited biosafety level 3 laboratories at Chang Gung Memorial Hospital or the National Defense Medical Center.

### Cell and culture medium

A549-ACE2-TMPRSS2 cells (designated as A549-OE) were purchased from InvivoGen (#20K23-NJ, San Diego, California) and maintained in DMEM with 10% heat-inactivated fetal bovine serum (FBS), 1% Penicillin/streptomycin (PS), 0.5 mg/mL of Puromycin and 300 mg/mL of Hygromycin B (InvivoGen) in 5% CO_2_ at 37℃. SF268 and Jurkat cells were purchased from the ATCC (American Type Culture Collection). SF268 cells were maintained in DMEM with 10% heat-inactivated FBS, 1% Penicillin/streptomycin/Amphotericin B (Gibco/Invitrogen). Jurkat cells were maintained in RPMI 1640 growth medium (Gibco/Invitrogen) supplemented with 10% heat-inactivated FBS (HyClone) and 1% PS (Gibco/Invitrogen).

Primary T-cells were isolated from fresh PBMCs using the EasySep Human T Cell Isolation Kit (negative selection method; STEMCELL Technologies, Cambridge, UK) according to the manufacturer’s instructions. PBMCs were isolated from healthy donors using Lymphoprep™ (Serumwerk Bernburg AG, Bernburg, Germany). Whole blood was diluted with an equal volume of PBS. Diluted blood samples (8 mL) were carefully layered onto 4 mL of Lymphoprep™ in a 15-mL centrifuge tube. After centrifuging at 800 ⋅ g for 20 min at 20 °C, PBMCs were carefully collected from the interface between the plasma and red blood cell layers. The collected PBMCs were washed with PBS, and red blood cells were lysed using ACK buffer (0.15 M NH_4_Cl, 10 mM KHCO_3_, and 0.1 mM Na_2_EDTA). The PBMCs were then processed for primary T-cell isolation. Primary T-cells were isolated using the EasySep Human T Cell Isolation Kit (STEMCELL Technologies) according to the manufacturer’s instructions. Briefly, an isolation cocktail was added to each sample and incubated at 25 °C for 5 min. RapidSpheres™ was then added and mixed gently, and the total volume was adjusted to 1.5 mL with the recommended medium (PBS containing 2% FBS and 1 mM EDTA). The tube was placed on a magnet for 3 min, and primary T-cells were collected from the supernatant. The purity of the isolated primary T-cells was confirmed to be > 90% using the PE anti-human CD3 antibody (BioLegend, San Diego, CA, USA). The protocol for PBMC isolation was approved by Chang Gung Memorial Hospital (IRB number # 202001041A3C).

## Cell surface expression of ACE2, TMPRSS2, or HLA-C

Cells were suspended in staining buffer (PBS containing 1% skim milk) at a density of 1 × 10^5^ cells per sample and incubated with anti-ACE2 (1: 500, R&D Systems), anti-TMPRSS2 (1: 250, Santa Cruz Biotechnology), and anti-HLA-C (1:500, Novus Biologicals) antibodies at 4 °C for 30 min. After washing with PBS, FITC-conjugated secondary antibodies (Thermo Fisher Scientific, Waltham, MA, USA and Jackson Immunoresearch, West Grove, PA, USA) at 1 µg per sample were added and incubated at 4 °C for 30 min. The detailed information about the primary and secondary antibodies is shown in Table S2. Cell surface expression of ACE, TMPRSS2 or HLA-C was analyzed using a flow cytometer (BD Accuri C6). A defined gating strategy was applied to identify the target cell populations shown in Fig. S4, and 10,000 events were acquired per sample. Background fluorescence and gate settings were determined using appropriate isotype controls (Table S2). According to the secondary antibodies conjugated with fluorescence, e.g., ACE2, were analyzed using the FL1 (AF488) channel. Representative flow cytometry plots illustrating the gating strategy and data quality have been added to Fig. S4.

### Viral infection in A549-OE, SF268 and T cells

Cells were seeded at a density of 1× 10^4^ cells per well in 96-well microculture plates. Subsequently, S protein (Alpha, BA.1, BA.4/5, and XBB) pseudotyped lentiviruses (MOI = 0.5) were added and incubated for 24 h. The culture medium was then replaced with fresh growth medium, and cells were incubated for an additional 48 h. Infectious cells were collected and transferred to a white plate. A culture medium containing d-luciferin (Perkin Elmer, Waltham, MA, USA) at a final concentration of 150 mg/mL was added. Luminescence (Relative Light Units, RLU) was measured using a BioTek Synergy H1 microplate reader.

### MH-1 competition with SARS-CoV-2 S-pseudotyped lentivirus infection in A549-OE, SF268, and T cells

Cells at a density of 1×10^4^ cells/well were seeded in 96-well microplates and MH-1 peptide was added simultaneously to each S-pseudotyped lentivirus (MOI = 0.5) and incubated for 24 h. The culture medium was replaced with fresh growth medium, and cells were incubated for an additional 48 h. Cells were collected and added to a white plate on which a culture medium supplemented with d-luciferin (Perkin Elmer) was added at a final concentration of 150 mg/mL. Luminescence (RLU) was measured using a luminescence filter on a BioTek Synergy H1 microplate reader.

### HLA-C knockdown in Jurkat cells with shRNA virus

Lentiviral particles with the lentiviral vector expressing HLA-C shRNA (#333639, #353148, and #363747) and scrambled shRNA were purchased from the RNA Technology Platform and Gene Manipulation Core (Academia Sinica, Taipei, Taiwan). Three target sequences for HLA-C shRNA lentiviral constructs are 5′-GCAACTTCTTACTTCCCTAAT-3′ (#333639), 5′-AGCTGTGGTCACCGCTATGAT-3′ (#353148), and 5′-GCAGAGATACACGTGCCATAT-3′ (#363747), respectively. The target sequence for the scrambled shRNA sequence is 5′-CCTAAGGTTAAGTCGCCCTCG-3′. Jurkat cells were seeded at a density of 2 × 10^5^ cells per well in 6-well plates. Subsequently, HLA-C shRNA lentiviruses (MOI = 5) were added to the plates and incubated for 24 h. The supernatant was then discarded, and fresh culture medium containing 2 µg/mL puromycin was added for selection. After 6 days, the puromycin-selected Jurkat cells were analyzed by q-PCR and flow cytometry to assess the knockdown efficiency of the HLA-C gene and surface expression (Fig. S5). The puromycin-selected Jurkat cells were then used for subsequent experiments.

Knockdown efficiency of three HLA-C shRNA lentiviral constructs was evaluated at both the mRNA and protein levels. These three shRNA constructs reduced HLA-C mRNA expression by approximately 96.7% (#333639), 70.9% (#353148), and 72.8% (#363747), respectively. They also reduced HLA-C protein expression by approximately 93.9% (#333639), 55.1% (#353148), and 67.4% (#363747), respectively. Among them, shRNA #333,639 demonstrated the highest knockdown efficiency and was selected for the subsequent viral infection.

### Effect of HLA-C knockdown in Jurkat cells on SARS-CoV-2 infection

Jurkat and puromycin-selected Jurkat cells (with the #333639 shRNA knockdown) were seeded at 1×10^4^ per well in 96-well and pretreated with an ACE2 neutralizing antibody (1 µg/mL, Cat#10108-MM37, Sino Biological) and a TMPRSS2 inhibitor (1 µM Camostat mesylate, Selleck Chemicals, USA) for 30 min, then infected with SARS-CoV-2 S-pseudotyped lentivirus (Alpha, BA.1, BA4/5, and XBB) or control virus (Ctrlv) at MOI 0.5 were added and incubated for 24 h. The culture medium was then replaced with fresh growth medium and cells were incubated for an additional 48 h. Infectious cells were collected and transferred to a white plate. The culture medium containing d-luciferin (Perkin Elmer, Waltham, MA, USA) at a final concentration of 150 mg/mL was added. Luminescence (Relative Light Units, RLU) was measured using a BioTek Synergy H1 microplate reader. Samples were collected 48 h post-infection. Data from samples are based on four replicates (*n* = 4).

### SARS-CoV-2 plaque reduction assay

VeroE6 cells were seeded at a density of 1.2 × 10^6^ cells in a 6-well plate 1 day before infection. The virus (50 plaque-forming units, PFU) was mixed with varying concentrations of the MH-1 peptide and added to the cells. After 1 h of adsorption at 35 °C, cells were washed with PBS and supplemented with 3 mL of 1.2% microcrystalline cellulose (MCC; 435244, Sigma-Aldrich) in serum-free DMEM. Cells were incubated at 35 °C for 72 h, then fixed with 10% formaldehyde. Subsequently, cells were stained with crystal violet, and viral plaques were counted and calculated as PFU per milliliter. 

### Investigation of the effects of MH-1 on the SARS-CoV-2 pseudovirus’ pulmonary uptake via whole-animal luminescent imaging

Female hACE2 homo (C57BL/6JNarl) mice (> 31-week-old) were purchased from Charles River Laboratories (Frederick, Maryland, USA). SARS-CoV-2-S Luc pseudo-typed lentivirus (bearing B.1.1.7 Alpha S-protein, 3.8 × 10^4^ RIU) was incubated respectively with 10 µM MH-1 and mCherry protein (control) for 1 h at room temperature. Thereafter, the MH-1-pseudovirus mixture and the mCherry-pseudovirus mixture were administered intranasally to the mice in the treatment and control groups (*n* = 4–5), respectively. The mice were then incubated for one day to allow for pulmonary uptake of the pseudovirus. At the end of the incubation, the mice were administered intraperitoneally with 150 mg/kg of D-luciferin (GoldBio). Then, they were anesthetized with isoflurane using a precision vaporizer and oxygen at 3 min post D-luciferin administration. Whole-animal luminescent imaging was subsequently performed (using 8 binning with a F-stop of 1, and exposure time: 10 min). To quantify the luminescence signals from the mice, the chest region in the displayed images was quantified as total radiance (photon) using ROI tools by IVIS Series 2000 (PerkinElmer). All luminescent signals collected were normalized and reported as photons/second/cm^2^/sr. All procedures were approved by the Institutional Animal Care and Use Committee of Chang Gung University, Taiwan.

### Investigation of the effect of MH-1 on SARS-CoV-2 infection in K18-hACE2 mice

Omicron sublineages, such as XBB.1.1, have accumulated more RBD mutations than Alpha, Beta, or Delta strains, resulting in significant antigenic differences. Therefore, XBB.1.1 was chosen for antiviral evaluation. To evaluate the inhibitory efficacy of MH-1 on viral infection, 12–15-week-old female K18-hACE2 mice (purchased from Jackson Laboratory) were used. A total of 80 µL of either mCherry or MH-1 protein solution (2 mg/kg) was mixed with 20 µL of the XBB. 1.1 virus (34 000 PFU) and incubated for 1 h at 25 °C. Subsequently, 30 µL of the MH-1–SARS-CoV-2 or mCherry–SARS-CoV-2 virus mixture was intranasally administered to the treatment and control groups (*n* = 3), respectively. Mice were euthanized on day 4 post-inoculation via intraperitoneal (IP) injection of a pentobarbital overdose to ensure a rapid and painless death. The right lung was homogenized for viral quantitative PCR, while the left lung was harvested for histopathological staining. Experiments involving infectious SARS-CoV-2 were conducted in Institutional Biosafety Committee-approved BSL3 and A-BSL3 facilities at the National Defense Medical Center. Animal studies were conducted in accordance with the Animal Research: Reporting of In Vivo Experiments guidelines.

### Lung viral load quantification

Lung tissue samples were homogenized using Precellys bead disruption (CK14 lysing kit) in DNA/RNA shield (Zymo) and centrifuged at 10,000 rpm for 5 min to pellet cell debris. RNA was extracted from the supernatant using a LabTurbo AIO Viral DNA/RNA Extraction Kit (Taigen Bioscience) according to the manufacturer’s instructions. To detect viral RNA, 5 µL of RNA was used with the TaqMan Fast Virus 1-Step Master Mix (Applied Biosystems) targeting the N gene of SARS-CoV-2. RT-qPCR was performed on a LabTurbo AIO 48 SP-qPCR System (Taigen Bioscience). The primer sequences are shown in Table S3. Dilutions of in vitro transcribed RNA standards (including the viral N gene and mouse actin gene) were quantified using droplet digital PCR (Qiagen). These standards were used to calculate the number of target RNA copies. The relative mRNA expression levels were calculated using the 2^−ΔΔCq^ method.

### Histology and immunohistochemistry

Lung tissues were fixed in 4% formaldehyde for at least 7 days to ensure complete viral inactivation, then embedded in paraffin. Tissue Sect. (3 μm thick) were prepared and stained with hematoxylin and eosin (H&E). IHC analyses were performed as follows: serial paraffin sections were first deparaffinized using Tissue-Tek DRS 2000 (SAKURA). After blocking endogenous peroxidase activity and performing antigen retrieval, the slides were incubated overnight with anti-SARS-CoV-2 nucleocapsid antibodies (1:10000, 40143-T62; Sino Biological). Subsequently, the slides were treated with donkey anti-rabbit IgG (1:200, 711-035-152; Jackson ImmunoResearch) for 3 h. Labeling detection was carried out using the DAB Substrate Kit (SK-4100; Vector Laboratories), according to the manufacturer’s protocol. The sections were then counterstained with hematoxylin (GHS216, Sigma-Aldrich). Finally, the slides were immediately digitized using the Olympus VS200 system.

### Statistical analysis

Data presented in Figs. 3 and 4 were statistically analyzed using one-way analysis of variance (ANOVA) followed by Dunnett’s post hoc test. The nonlinear regression models with variable slopes were employed to assess the IC_50_ of MH-1 in inhibiting SARS-CoV-2 infection. The unpaired, two-tailed Student’s *t*-test was used to compare the differences between the MH-1 group and the control group in the percentage of plaque reduction at the same concentration and in the animal study. Statistical analyses were conducted using GraphPad Prism v.9.3.3 (GraphPad Software, Inc., CA, USA). Data are presented as the mean ± standard deviation (SD) from at least three independent biological replicates, where data in statistical significance was set at *: *p* < 0.05; **: *p* < 0.01; ***: *p* < 0.001.

## Results

### The MH-1 peptide sequence and its predicted binding to the SARS-CoV-2 S-RBD

**Fig. 1:**
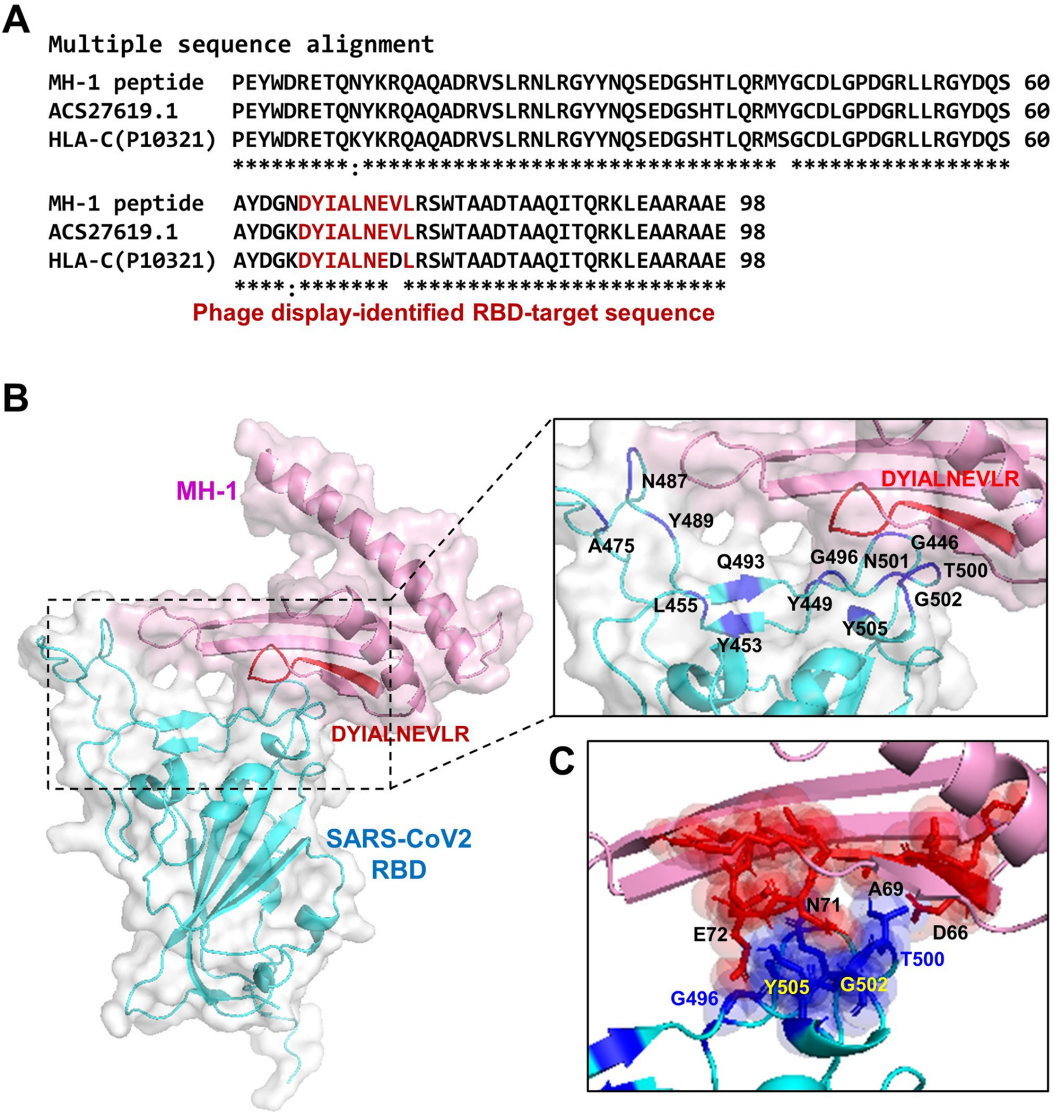
The MH-1 peptide sequence and its predicted binding to the SARS-CoV-2 S-RBD. The phage display shows that the “DYIALNEDLR” peptide sequence bound to the SARS-CoV-2 S-RBD is derived from HLA-C. **A** Alignment of the MH-1 peptide sequence identified in this study with the human MHC 1 (ACS27619.1) and HLA-C (Uniprot ID: P10321). The red-highlighted region indicates the specific SARS-CoV-2 S-RBD binding site identified through phage display screening. **B** Protein-peptide docking predicted the binding position of MH-1 peptide (pink) to SARS-CoV-2 S-RBD (cyan). The 3D structure of MH-1 peptide was modeled using the SWISS-MODEL web tool. MH-1 was docked to the SARS-CoV-2 S-RBD (PDB: 8XYZ, chain A) using HADDOCK2.4. MH-1 binds to the RBD and blocks most of the residues that can bind to ACE2. **C** The “DYIALNEDLR” peptide interacted with SARS-CoV-2 RBD at G496, T500, G502, and Y505 (blue)

To obtain an effective inhibitory peptide, we successfully screened candidate peptides that target the receptor-binding domain of the SARS-CoV-2 S-RBD using phage display. In addition to peptides derived from ACE2 and TLR4 receptors, a peptide, DYIALNEDLR, also showed high affinity. Subsequently, the sequence alignment of the peptide “DYIALNEDLR” with those archived in the National Center for Biotechnology Information (NCBI) database was performed using CLUSTAL O (version 1.2.4). This multiple sequence alignment identified the peptide as a component of the extracellular domain of the major histocompatibility complex class I (MHC-I), specifically HLA-C (Fig. 1A and Fig. S6A). This peptide also shares a highly conserved sequence with the HLA-C sequence (P10321, Fig. 1A). A partial MHC-I peptide containing the DYIALNEDLR sequence (termed “MH-1”) was then designed (Fig. 1A), and its ability to bind to the SARS-CoV-2 RBD was predicted. The 3D structure of the MH-1 peptide was modeled using the SWISS-MODEL web tool. MH-1 was docked to the SARS-CoV-2 S-RBD (PDB: 8XYZ, chain A) using HADDOCK24. MH-1 binds to the RBD and blocks most of the residues that can bind to ACE2 (Fig. 1B). The docking result showed that MH-1 binds to the RBD and blocks most of the residues that typically interact with ACE2 (Fig. 1B). Notably, the peptide “DYIALNEDLR” strongly interacts with the SARS-CoV-2 RBD at residues G496, T500, G502, and Y505 (Fig. 1C). The MH-1 peptide potentially blocks the interaction between host ACE2 and the SARS-CoV-2 S-RBD by binding to the RBD.

After predicting the potential for binding to the RBD, the MH-1 peptide was expressed recombinantly (Fig. S6B) and characterized by anti-HLA-C monoclonal antibodies (Fig. S6C). This synthesized MH-1 was used as an inhibitor of the interaction between ACE2 and the S protein receptor binding domain (S-RBD) of SARS-CoV-2 in subsequent experiments. The mCherry peptide was utilized as a control peptide.

### Investigation of MH-1 interference on the S-RBD–ACE2 interaction using an electrochemical impedance spectroscopy-based biosensing platform

To investigate whether the MH-1 peptide interferes with the S-RBD–ACE2 interaction, our research team established a biosensing platform based on electrochemical impedance spectroscopy (EIS) to monitor electrical signaling. We first immobilized the MH-1peptide onto the palladium nanothin film (Pd-NTF) electrode of the biosensing platform and exposed the MH-1-coated electrode to escalating concentrations of omicron S-RBD suspended in phosphate-buffered saline (PBS). An increase in biosensor impedance (represented by an increase in the Rct value, ΔRct) indicated binding of S-RBD to the immobilized MH-1, whereas a reduction in ΔRct suggested interference with the binding.

**Fig. 2:**
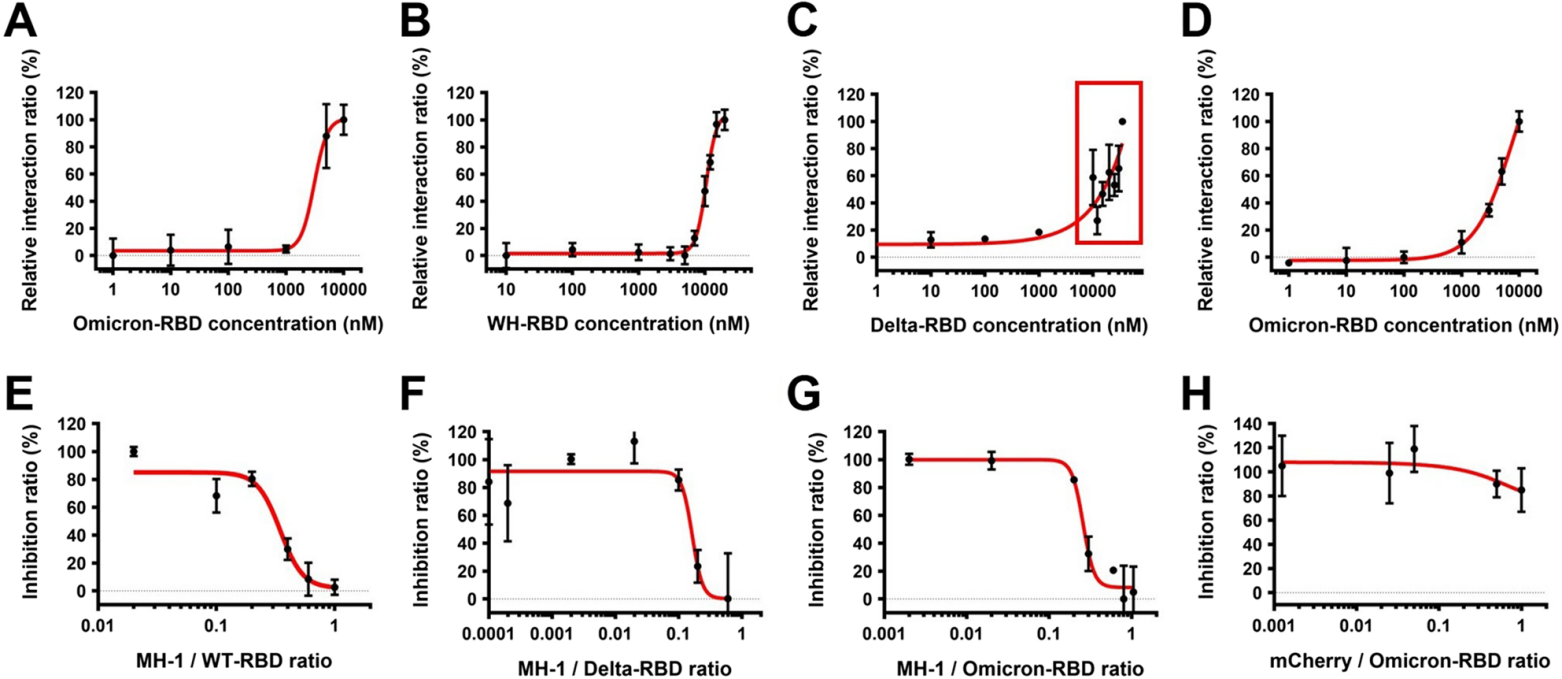
Relative impedance changes in Pd-NTF electrodes in response to SARS-CoV-2 RBD variants and MH-1 peptide. Relative impedance changes ratios (∆Rct ratio) in response to the exposure of (**A**) MH-1-coated Pd-NTF electrode to increasing concentrations of the Omi-RBD (BA.1). ∆Rct ratio in response to the exposure of the ACE2-Pd-NTF electrode to increasing concentrations of the S-protein receptor-binding domain (S-RBD) of SARS-CoV-2: (**B**) Wuhan, (**C**) Delta, and (**D**) Omicron (BA.1) variants. The red box highlights that the Delta RBD leads to reduced RBD-ACE2 binding due to aggregation at high concentrations, preventing the plotting of the curve. ∆Rct ratio in response to the exposure of the ACE2-Pd-NTF electrode to MH-1-S: (**E**) Wuhan RBD, (**F**) Delta RBD, and (**G**) Omi-RBD (BA.1) variant at increasing MH-1 concentrations. **H** ΔRct ratio of ACE2-Pd-NTF electrodes exposed to mCherry control-S Omi-RBD (BA.1) variants at increasing mCherry concentrations (generated using the biosensing platform). Error bars indicate standard deviation (SD)

We observed a concentration-dependent increase in relative ΔRct ratio following the exposure of the MH-1-coated biosensing electrode to increasing concentrations of Omi-RBD, thereby confirming the binding of MH-1 to Omi-RBD (dissociation constant, Kd = 10.12 ± 0.63 µM) (Fig. 2A). Using the biosensing platform, recombinant ACE2 protein was immobilized on a Pd-NTF electrode, and different concentrations of Wuhan RBD (WH-RBD), Delta RBD, and Omi-RBD (BA.1 variant) were added (Fig. 2B–D). The binding affinities of ACE2 to WH-RBD and Omi-RBD were calculated to be Kd = 9.44 ± 0.78 µM and Kd = 4.32 ± 0.3 µM, respectively. In contrast, the Delta RBD, due to its aggregation at high concentrations (indicated by the red box), showed reduced RBD-ACE2 binding, thereby making it impossible to calculate its Kd value (Fig. 2C). To assess the competitive binding capabilities of MH-1, we premixed varying concentrations of MH-1 with WH-RBD, Delta RBD, Omi-RBD, or mCherry control peptide at 25 °C for 10 min (Fig. 2E–H). The mixtures were then added to Pd-NTF electrodes with immobilized recombinant ACE2, and ΔRct was measured. MH-1 inhibited the binding of S-RBD (Wuhan, Delta, and Omicron variants) to ACE2 in a concentration-dependent manner, as reflected by a reduction in ΔRct ratio with increasing MH-1 concentration. The IC_50_ values were 0.3 mg/mL (7.9 µM) for WH-RBD, 0.15 mg/mL (3.9 µM) for Delta-RBD, and 0.25 mg/mL (6.6 µM) for Omi-RBD (Fig. 2E–G). In contrast, the control peptide showed no change in ΔRct ratio regardless of concentration, which indicated that the control peptide did not affect Omi-RBD binding to ACE2 (Fig. 2H). These results demonstrated that MH-1 bound to free S-RBD of all variants and effectively attenuated their interactions with immobilized ACE2, suggesting that MH-1 may block SARS-CoV-2 infection by inhibiting S-RBD binding to ACE2.

### The MH-1 peptide inhibited the pseudotyped lentivirus infection in different cells

**Fig. 3:**
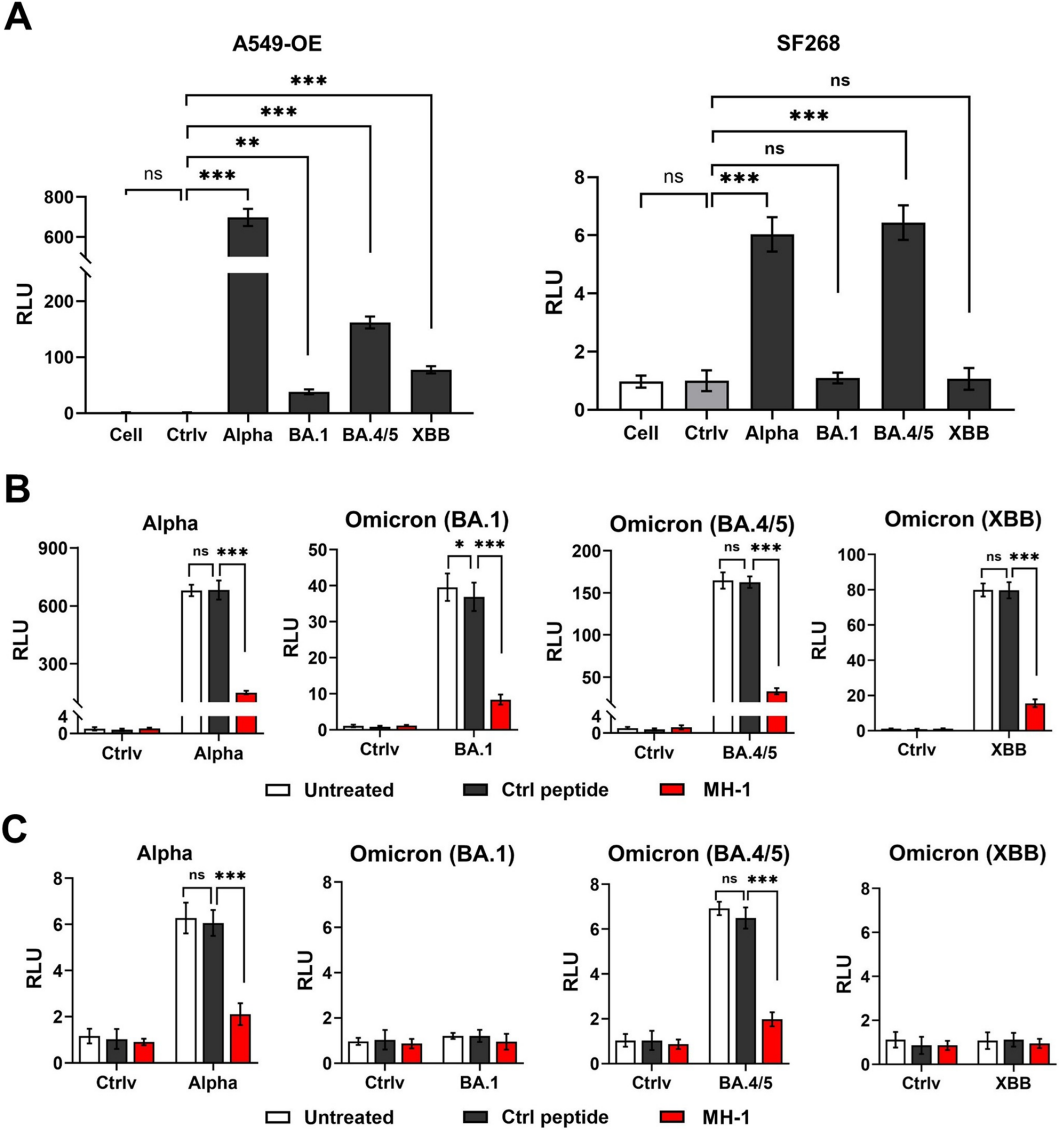
MH-1 peptide inhibits SARS-CoV-2 infection in A549-OE, and SF268 cells. **A** A549-OE and SF268 cells were infected with SARS-CoV-2 S-pseudotyped lentivirus, including Alpha, Omicron (BA.1), Omicron (BA.5), and Omicron (XBB) or control virus (Ctrlv, VSV-G) at MOI 0.5. Samples were collected 48 h post-infection. *: *p* < 0.05; ***: *p* < 0.001, compared with the Ctrlv group. Infection efficiency following MH-1 peptide or control peptide treatment with SARS-CoV-2 S-pseudotyped lentivirus on (**B**) A549-OE and (**C**) SF268 cells. Data for analysis were based on three replicates (*n* = 3). A549-OE: A549 cells with ACE2 and TMPRSS2 overexpression. Ctrl peptide: control peptide. Each dot represents one subject. ***: *p* < 0.001, compared with the Ctrl peptide group

To validate the S-RBD binding activity of the MH-1 peptide detected by the EIS biosensing platform, the SARS-CoV-2-S Luc-pseudotyped lentivirus was used to infect two cell lines: A549-OE cells (human non-small cell lung cancer cells that overexpress ACE2 and TMPRSS2, Fig. S4) and SF268 (human glioblastoma cells that do not express ACE2 and TMPRSS2, Fig. S4). Before evaluating the inhibitory effects of MH-1 peptides on SARS-CoV-2 infection, we confirmed the infectivity of four SARS-CoV-2 S-pseudotyped lentiviruses, such as B.1.1.7 (Alpha), BA.1.1.529 (BA.1, Omicron), BA.4/5 (Omicron), and XBB.1.16 (Omicron) (Table S1). These SARS-CoV-2 S-pseudotyped lentiviruses successfully infected A549-OE cells. Among the four pseudoviruses, Alpha was the most effective, followed by BA.4/5 (Fig. 3A). The pseudoviruses displayed low infectivity in SF268 cells. The Alpha remained the most infectious, followed by BA.4/5 (Fig. 3A), while BA.1 and XBB were nearly non-infective in SF268 cells.

The inhibitory effect of the MH-1 peptide on viral infection was then investigated. The MH-1 peptide did not cause any cytotoxicity to A549-OE and SF268 cells at the tested concentrations (Fig. S7). The MH-1 peptide significantly reduced the infectivity of these four pseudoviruses in A549-OE (Fig. 3B) and SF268 cells (Fig. 3C), while the control peptide had no effect. These results demonstrate that the binding of the MH-1 peptide to the SARS-CoV-2-S RBD effectively hinders viral infection.

### MH-1 peptide suppressed SARS-CoV-2 S-pseudotyped lentivirus infection in T cells

**Fig. 4:**
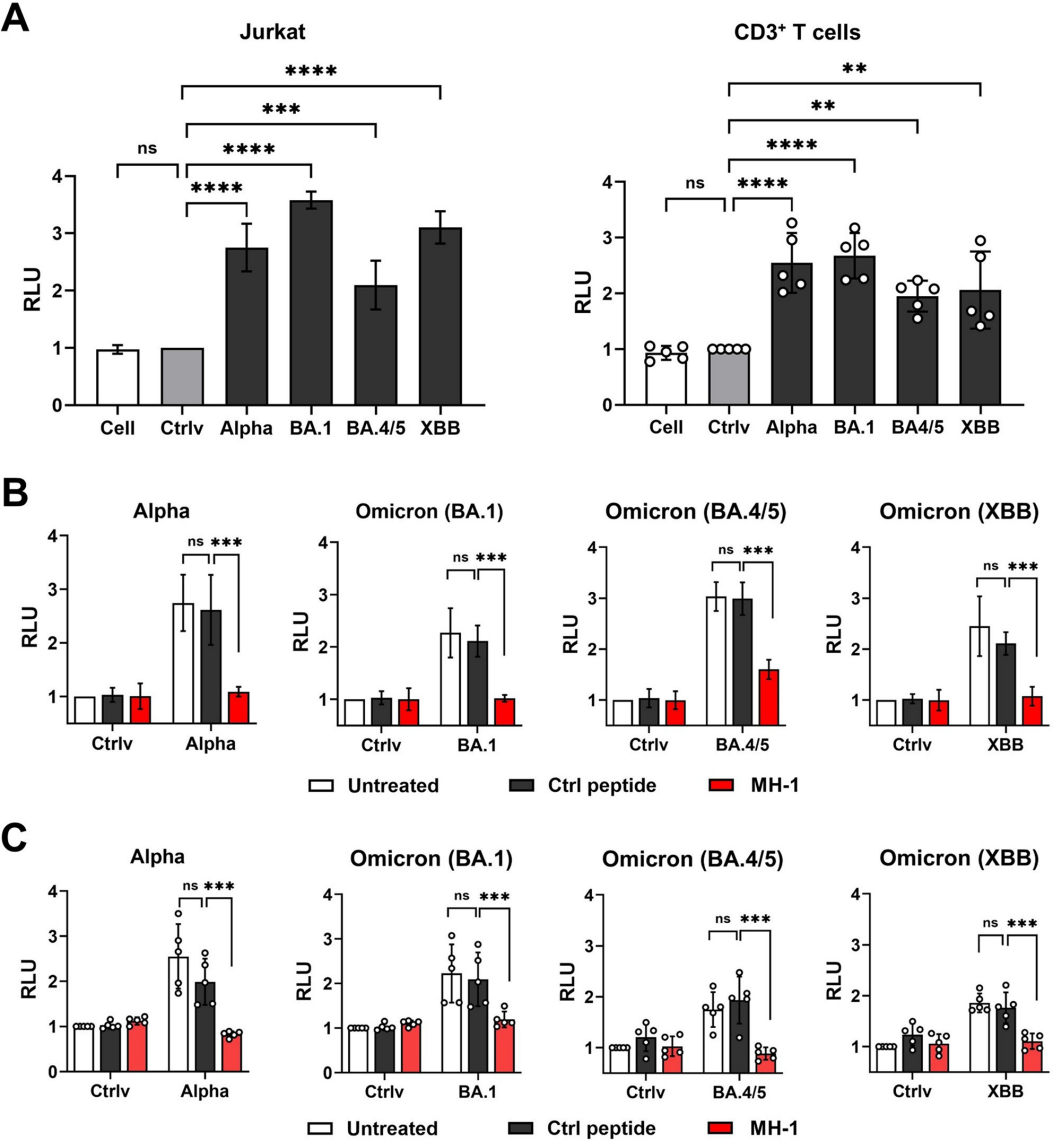
MH-1 peptide decreases SARS-CoV-2 S-pseudotyped lentivirus infection on T-cells. **A** Jurkat and primary T cells were infected with four SARS-CoV-2 S- pseudotyped lentiviruses, Alpha, Omicron (BA.1), Omicron (BA.5), and Omicron (XBB) or control virus (Ctrlv, VSV-G) at MOI 0.5. Samples were collected 48 h post-infection. **: *p* < 0.01; ***: *p* < 0.001; ****: *p* < 0.001, compared with the Ctrlv group. Infection efficiency following MH-1 peptide or control peptide treatment on SARS-CoV-2 S-pseudotyped lentivirus on (**B**) Jurkat and (**C**) Primary T cells. Primary T cells were isolated from healthy donors (*n* = 5). Ctrl peptide: control peptide. Each dot represents one subject. ***: *p* < 0.001, compared with the Ctrl peptide group

Recent studies have shown that SARS-CoV-2 can infect immune cells (particularly T cells), thereby diminishing their ability to combat the virus. We further investigated whether the MH-1 peptide could inhibit SARS-CoV-2 infection in immune cells. Jurkat (immortalized human T) and primary T cells were indeed infected by four types of pseudoviruses, even though they did not express ACE2 and TMPRSS2 (Fig. 4A). Furthermore, the variability in infection efficiency of different SARS-CoV-2 variant pseudoviruses observed in T cells may reflect the variant-specific spike protein properties and non-classical viral invasion pathways in ACE2-low-expressing immune cells. Despite these differences, the MH-1 peptide still exhibited excellent inhibitory effects against pseudovirus infection, effectively reducing T cell infection, with similar results across all four pseudoviruses (Fig. 4B, C). These results indicated that the MH-1 peptide directly binds to the RBD, allowing it to inhibit viral infections that occur through ACE2 and TMPRSS2-independent pathways.

The MH-1 peptide, derived from HLA-C, demonstrates a strong binding affinity for the SARS-CoV-2 S protein (Figs. 1B and 2). Additionally, T lymphocytes lack ACE2 and TMPRSS2, but exhibit higher expression levels of HLA-C (Fig. S4). These results led us to hypothesize that SARS-CoV-2 might bind to HLA-C and subsequently enter T lymphocytes. To test the hypothesis that SARS-CoV-2 can enter T-cells via HLA-C, we silenced HLA-C using HLA-C-specific shRNA in Jurkat cells and then evaluated the infectivity of SARS-CoV-2 pseudoviruses. After confirming the efficiency of HLA-C downregulation through gene expression and cell surface expression, HLA-C knockdown Jurkat cells were infected with the SARS-CoV-2 pseudoviruses. Downregulation of HLA-C significantly reduced the infectivity of SARS-CoV-2 pseudoviruses (Fig. S8). This suggests that HLA-C may contribute to viral susceptibility for SARS-CoV-2 to facilitate viral infection in T cells that lack ACE2 and TMPRSS2.

### The MH-1 peptide can inhibit infection by different SARS-CoV-2 variants

**Fig. 5:**
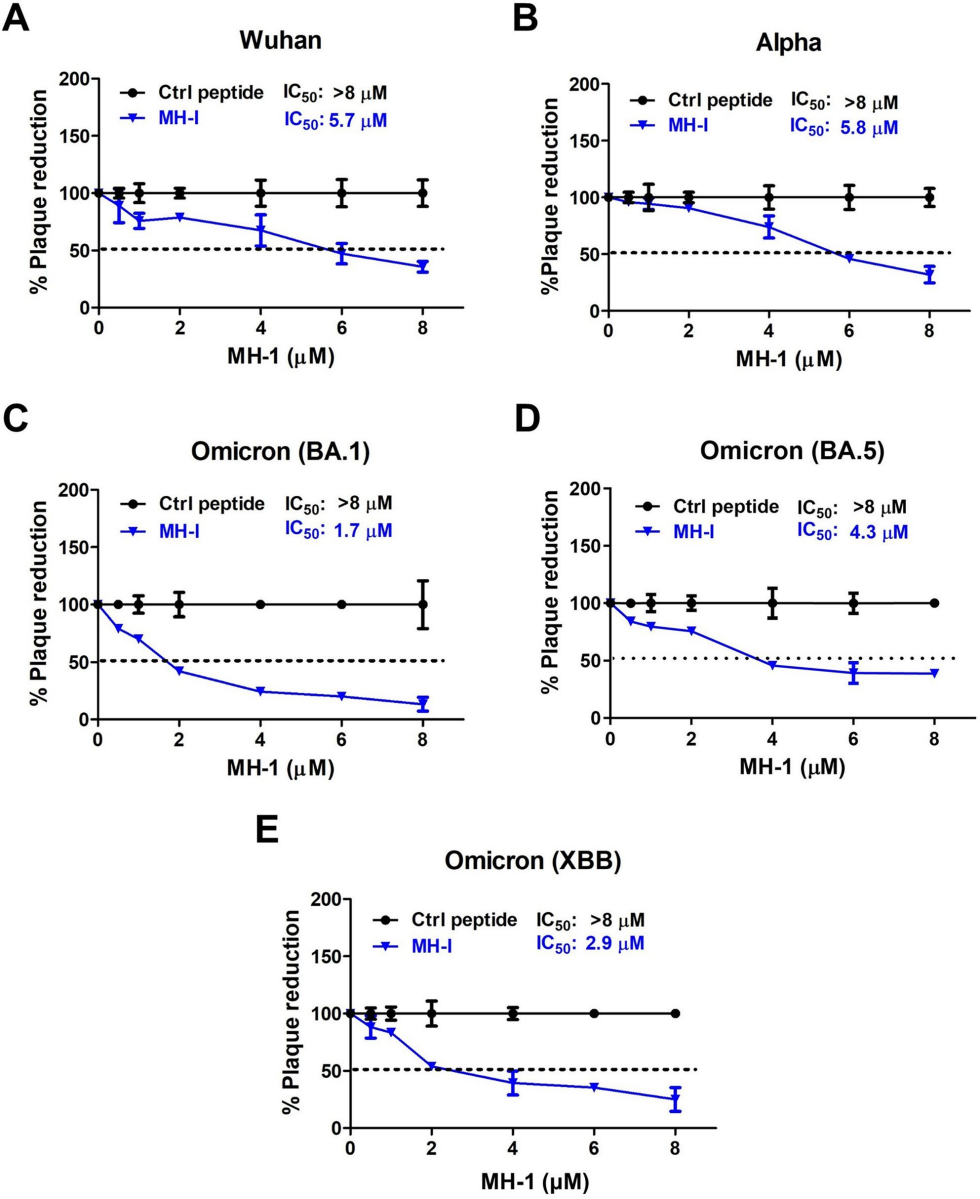
SARS-CoV-2 variants are inhibited by MH-1 peptide in VeroE6 cells. Vero E6 cells were infected with SARS-CoV-2 variants: (**A**) Wuhan, (**B**) Alpha, (**C**) Omicron (BA.1), (**D**) Omicron (BA.5), and (**E**) Omicron (XBB) (MOI = 0.005), in the presence of varying concentrations of MH-1 peptide or control peptide. After 1 h of adsorption at 35 °C, cells were washed and incubated for 3 days at 35 °C. Plaque numbers were normalized to the untreated group and expressed as a percentage. IC50 values for MH-1 in inhibiting SARS-CoV-2 were determined using nonlinear regression models with variable slopes. The data represent the results of three independent experiments. Ctrl peptide: control peptide

We conducted a plaque reduction neutralization test to evaluate whether the MH-1 peptide could disrupt the interaction between SARS-CoV-2 and host cells. The MH-1 peptide demonstrated significant inhibitory effects against infections caused by various SARS-CoV-2 variants, including the Wuhan, B.1.1.7, BA.1, and BA.4/5 variants, in VeroE6 cells at a concentration of 8 µM, achieving over 50% plaque reduction (Fig. 5). Notably, among the variants tested, the MH-1 peptide showed the most potent inhibitory effect against the BA.1 variant, with an IC_50_ of 1.795 µM (Fig. 5C), followed by the XBB variant with an IC50 of 2.9 µM (Fig. 5E). The IC_50_ of MH-1 peptide against other variants, including Wuhan, B.1.17, and BA.4/5 variants, is approximately 4.3–5.8 µM (Fig. 5A, B and D).

### MH-1 attenuates SARS-CoV-2 pseudovirus pulmonary uptake in vivo

**Fig. 6:**
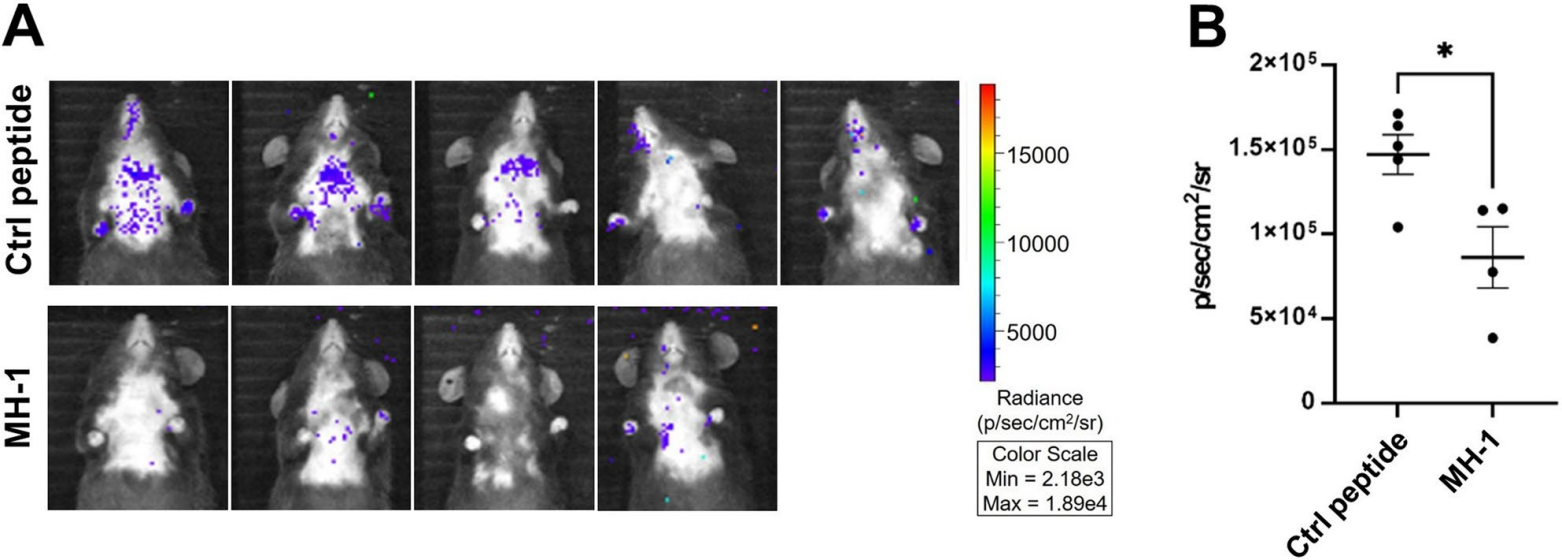
MH-1 attenuates the SARS-CoV-2 pseudovirus pulmonary uptake in vivo. **A** SARS-CoV-2-S Luc pseudo-typed lentiviruses (B.1.1.7) were actively taken up by the lungs after pre-treatment with the control peptide and the MH-1 peptide; (**B**) Comparison of the pseudovirus pulmonary uptake between mice groups receiving the control peptide or MH-1-pretreated pseudovirus (*: *p* < 0.05)

The in vivo virus inhibition capability of MH-1 using whole-animal luminescent imaging to assess the attenuating effects of MH-1 on the pulmonary uptake of the SARS-CoV-2 pseudovirus. To determine the susceptibility of hACE2 mice to pseudoviruses expressing different variants of the SARS-CoV-2 S protein, we intranasally injected hACE2 mice with various S-variant pseudoviruses. After 24 h, we measured the luminescence signal and observed that the pseudovirus with the B.1.1.7 spike variant exhibited the highest sensitivity in the mice (Fig. S9). This result is consistent with our previous cell experiment results, which show that B.1.1.7 exerts a more significant inhibitory effect against viruses than the other variants. Therefore, this specific variant was selected for further investigation in subsequent animal studies. A significant reduction (41.5%) in the pulmonary uptake of pseudovirus was observed in mice inoculated with the MH-1-pretreated pseudovirus compared with that of the control peptide-pretreated pseudovirus (Fig. 6). This suggests that MH-1 exerts a neutralizing effect against the pseudovirus in vivo.

#### MH-1 peptide reduces SARS-CoV-2 lung infection in K18-hACE2 mice

**Fig. 7:**
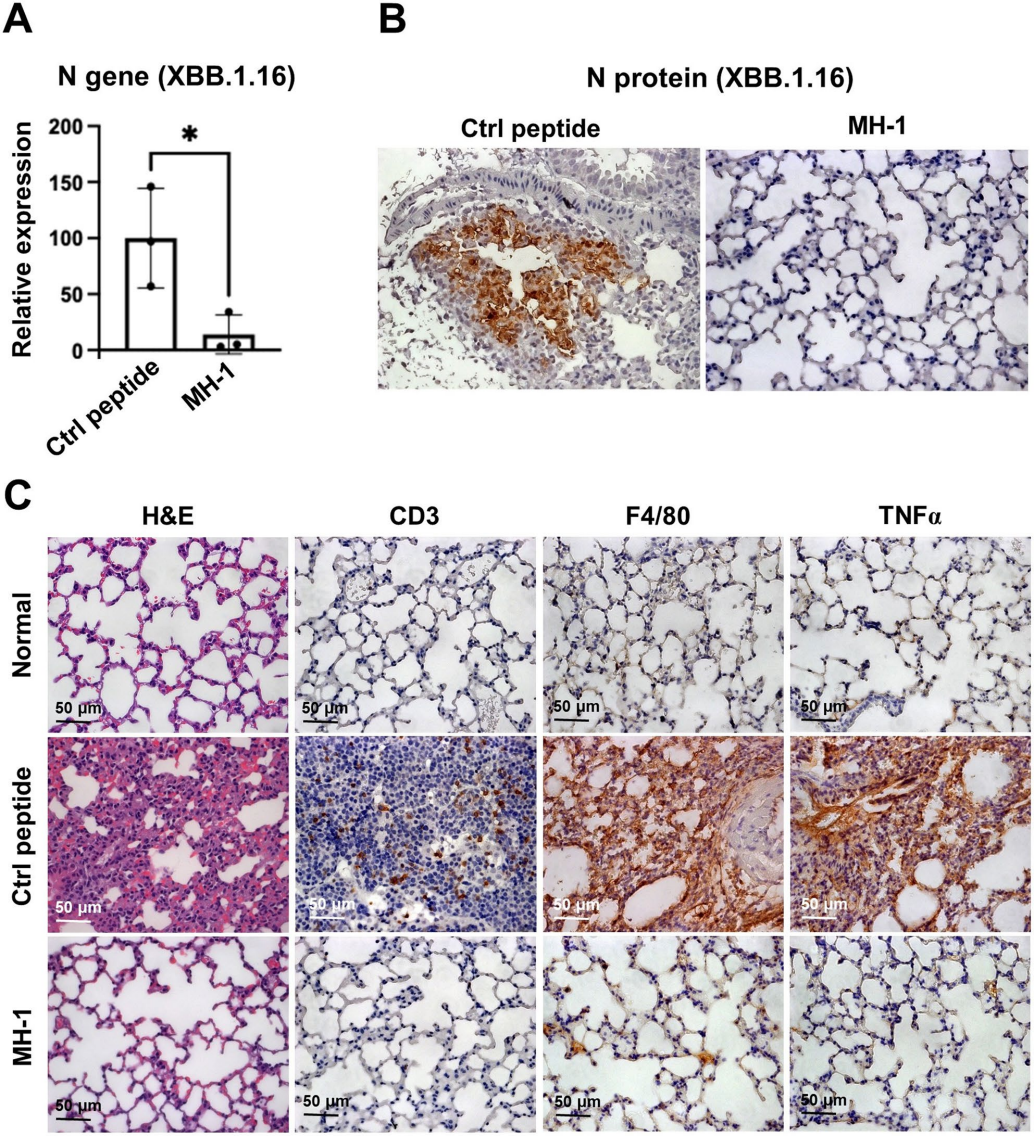
Intranasal administration of MH-1 or control peptide with XBB. 1.1 SARS-CoV-2 variant in K18-hACE2 mice. The control or MH-1 peptide (35.2 µM) and XBB. 1.1 variant of the SARS-CoV-2 virus (10,000 PFU) were incubated for 1 h at 25 °C. The MH-1–SARS-CoV-2 or control peptide–SARS-CoV-2 virus mixture was intranasally administered to K18-hACE2 mice in the treatment and control groups (*n* = 3), respectively. Lung tissue samples were collected on 4 dpi for (**A**) viral quantification by qPCR and (**B**) tissue section staining to assess viral expression levels and immune cell infiltration. Data are presented as the mean ± SD. Statistical significance was determined using an unpaired *t*-test (*: *p* < 0.05). **C** Lung histology and expression of CD3 (T cell), F4/80 (macrophage), and TNF-α in XBB. 1.1 SARS-CoV-2 variant-infected K18-hACE2 mice after treatment with MH-1

After demonstrating that MH-1 significantly inhibits infection by the SARS-CoV-2 pseudovirus with the Alpha B.1.1.7 variant in hACE2 mice, we next evaluated its effectiveness against the latest Omicron variant, XBB.1.16. In this experiment, the MH-1 peptide was preincubated with SARS-CoV-2 XBB. 1.16 for one hour before intranasal infection of K18-hACE2 mice. Four days post-infection (dpi), viral RNA levels in the lungs of the MH-1-treated group were significantly reduced by 86% compared to the control group (Fig. 7A). Histological analysis further supported this observation. Analysis of viral load in the lungs showed no or very low expression of N protein in mice treated with MH-1, while N protein expression was clearly detected in the lungs of mice treated with the control peptide (Fig. 7B). In the control peptide-treated mice, the alveolar wall thickness increased while the air space decreased. In contrast, mice treated with MH-1 showed no change in alveolar wall thickness, and their lung histology appeared similar to that of healthy lungs (Fig. 7C). CD3 and F4/80 staining demonstrated significant infiltration of T cells and macrophages in the control peptide-treated mice, whereas there was minimal immune cell infiltration in the MH-1-treated mice (Fig. 7C). Furthermore, mice treated with the control peptide exhibited increased TNF-α expression, while mice treated with MH-1 showed significantly lower TNF-α levels (Fig. 7C). Our results showed that the MH-1 peptide significantly inhibited viral infections and reduced immune cell infiltration and inflammation in the lungs in this small sample size of hACE2 mouse experiments. Future research requires larger sample sizes to validate and further develop these observations.

## Discussion

This study used an EIS biosensor to detect the inhibitory effect of the MH-1 peptide on SARS-CoV-2 infection and validated the inhibitory potential of the S-RBD-bound MH-1 peptide through cell and animal experiments. Palladium nano-thin-film electrodes have become effective biosensor substrates due to their high biological material binding efficiency and electrochemical sensitivity (Chang et al. 2019). We developed an ACE2-functionalized Pd-NTF EIS biosensor to monitor S protein-ACE2 interactions. This biosensor not only successfully identified the peptide inhibitor MH-1 (Fig. 2), which reduces viral binding and infectivity, but also serves as a screening tool for antiviral compounds (Kiew et al. 2021), indicating that this ACE2-functionalized Pd-NTF EIS biosensor can be flexibly used to detect SARS-CoV-2 antagonists, such as compounds, peptides, and even proteins.

Over the past 3 years, various SARS-CoV-2 inhibitors have been developed, including neutralizing antibodies, convalescent plasma (Izda et al. 2021; Jiang et al. 2020), recombinant human sACE2 (Zoufaly et al. 2020), ACE2-like decoy receptors (Linsky et al. 2020), S1 and S2 targeted peptides (Karoyan et al. 2021; Curreli et al. 2020), and small molecules inhibiting the ACE2:S1 RBD interaction (Chitsike and Duerksen-Hughes 2021). However, these inhibitors have limitations, including reduced efficacy against new variants (e.g., those harboring the E484K mutation), insufficient competitive binding to S1 RBD (Chitsike and Duerksen-Hughes 2021; Morgan et al. 2021), and the modest antiviral potencies of small molecules targeting ACE2:S1 RBD interactions, which require further optimization before being developed into efficacious agents (Chitsike and Duerksen-Hughes 2021; Day et al. 2021). Our study demonstrated that MH-1 exhibited high inhibition to various SARS-CoV-2 variants (Fig. 2) and effectively reduced infection in cells with varying ACE2 and TMPRSS2 expression levels (Figs. 3 and 5). Furthermore, MH-1 also protects T lymphocytes from various SARS-CoV-2 variant infections (Fig. 4B and C). MH-1 also effectively reduced infection in mice by both the B.1.1.7 alpha pseudovirus (Fig. 6) and the XBB virus (Fig. 7). These results demonstrate that MH-1 can prevent SARS-CoV-2 infection and exert blocking effects of MH-1 on early stages of viral infection, which are involved in the process of viral attachment or binding. Therefore, the observed inhibitory effect of MH-1 peptide reflects interference with early virus-host interactions.

The MH-1 peptide containing the “DYIALNEDLR” sequence exhibited high binding activity to SARS-CoV-2 RBD. Molecular docking showed the 3D structure and interaction between the MH-1 peptide and SARS-CoV-2 RBD. The MH-1 peptide effectively interacts with the RBD, and its binding residues coincide with those that engage with ACE2. On the RBD in Fig. 1B, residues K417, G446, Y449, Y453, L455, F456, A475, F486, N487, Y489, Q493, G496, Q498, T500, N501, G502, and Y505 are shown to form direct contacts with ACE2 (Lan et al. 2020; Shang et al. 2020; Wang et al. 2020). Among these residues, F486, Q493, and N501 are particularly important, as mutations at these sites markedly alter binding affinity and antibody recognition. For example, the Q493, Q498, and N501 mutations, seen in the Alpha and Omicron variants, enhance ACE2 affinity (Starr et al. 2020). Additionally, Q493 and K417 participate in key hydrogen bonds and salt bridges that stabilize the RBD-ACE2 interface. Our results showed that MH-1 bound to most of the RBD residues that interact with ACE2 (Fig. 1B, upper right), and the “DYIALNEDLR” fragment bound to the RBD at G496, T500, G502, and Y505 (Fig. 1B, lower right). Therefore, the binding response of MH-1 to RBD may be the reason why MH-1 exhibits excellent inhibition of SARS-CoV-2 infection.

HLA proteins play a vital role in presenting antigens to CD8 + T cells, thereby enabling immune surveillance. Expressed on nearly all nucleated cells (Wieczorek et al. 2017; Hewitt 2003), they are abundant in immune cells, particularly lymphocytes (Fig. S10). The MH-1 peptide, derived from HLA-C, has a strong binding ability for the SARS-CoV-2 S protein (Figs. 1B and 2E-G). These results further led us to hypothesize that SARS-CoV-2 might bind to HLA-C and subsequently enter infected T lymphocytes, given their high expression of HLA-C and the absence of ACE2 and TMPRSS2 (Fig. S4). All variants of SARS-CoV-2 can effectively infect both Jurkat T cells and primary T cells (Fig. 4B). When HLA-C was downregulated, the infectivity of all SARS-CoV-2 variants decreased (Fig. S8). These findings suggest that SARS-CoV-2 may infect T cells even when ACE2 and TMPRSS2 are expressed at low or undetectable levels, possibly involving HLA-C as a host factor contributing to viral susceptibility. While SARS-CoV-2 primarily replicates in respiratory tract cells, previous reports of viral particles in lymphocytes lacking phagocytic activity (Ren et al. 2021; Delorey et al. 2021) raised questions about its ability to infect T-cells. Furthermore, the interplay between SARS-CoV-2 and the immune system remains critical; the virus employs strategies such as downregulating MHC I expression in infected cells (Zhang et al. 2021; Yoo et al. 2021; Arshad et al. 2023), reducing CD8 + T-cell recognition (Agerer et al. 2021), and promoting viral replication. Interestingly, the variability in infection efficiency between different pseudoviruses in T cells was observed. Viral infection of T cells is highly dependent on variant-specific spike protein properties and alternative host factors (such as HLA-C), leading to significant differences in infection efficiency among different pseudoviruses. Our findings provide an alternative explanation that HLA-C facilitates viral infection into T-cells in the absence of ACE2.

Lymphocytopenia, a hallmark of severe COVID-19 cases, is characterized by damage to the spleen and hilar lymph nodes, lymphocyte apoptosis, and depletion of lymphoid tissue. SARS-CoV-2 RNA and coronavirus-like particles have been detected in lymphoid tissues through in situ hybridization assays and transmission electron microscopy in fatal cases (Xiang et al. 2021). Given the high levels of HLA-C expression in lymphoid-related tissues (e.g., appendix, colon, spleen, and lymph nodes; Fig. S10) and immune cells (e.g., T-cells, B-cells, dendritic cells, and monocytes; Fig. S10), it is plausible that HLA-C may act as a host factor facilitating viral infection, leading to lymphocyte destruction and lymphocytopenia. In addition to general cell entry receptors, several proteins specifically facilitate SARS-CoV-2 interactions with immune cells. For instance, SARS-CoV-2 infects T-cells via LFA-1 (Shen et al. 2022) and monocytes via the receptor for advanced glycation end products (RAGE) pathway (Angioni et al. 2023). Although SARS-CoV-2 can infect T cells through different pathways, MH-1 still effectively binds to the S protein (Figs. 1B and 2A) and reduces SARS-CoV-2 infection (Fig. 4B and C), demonstrating that MH-1 can protect immune cells from infection and prevent immune cell damage.

The K18-hACE2 mouse model, which is known to support infection with Omicron variants including XBB. 1.1, was selected for these in vivo experiments. These variants typically result in lower viral loads in the lungs, attenuated pulmonary pathology, and no significant body weight loss or mortality compared with the original Wuhan strain (Shuai et al. 2022; Chang et al. 2024). MH-1 was initially designed to disrupt the interaction between host ACE2 and the RBD of the SARS-CoV-2 (Wuhan) strain. As we know, Omicron variants, such as XBB.1.1, have accumulated more RBD mutations than Alpha, Beta, or Delta strains, resulting in significant antigenic differences. Therefore, XBB.1.1 was chosen for our antiviral evaluation. MH-1 is expected to inhibit infection for various SARS-CoV-2 variants due to its mechanism, but its antiviral efficacy against more pathogenic variants may require further evaluation of efficacy and duration.

Patients with COVID-19 carrying certain HLA-C alleles often experience severe clinical courses (Weiner et al. 2021; Sakuraba et al. 2020; Hovhannisyan et al. 2022). Moreover, SARS-CoV-2-induced methylation changes in HLA-C have been linked to dysregulation in HLA-C expression, which enables the virus to evade recognition by CD8 + T cells and enhances its survival in the host (Loi et al. 2022). Our observation that HLA-C facilitates viral infection in T cells may explain the higher infection rate and severity of disease in such patients. This mechanism may also contribute to the aggravation of SARS-CoV-2 infection in lung tissues, which express high levels of HLA-C (Fig. S10); and other body tissues with low or no ACE2 expression. MH-1 effectively inhibited SARS-CoV-2 infection in cells with varying HLA-C expression levels (Figs. 3B and C, 4B and C and 5), indicating that MH-1 can hinder SARS-CoV-2 infection in lung and immune cells with high HLA-C expression and demonstrating its inhibitory effectiveness.

## Conclusion

In this study, we used an EIS-based biosensor to rapidly detect the binding capacity and affinity of MH-1 for ACE2. Cell-based studies demonstrated that MH-1 binds to the SARS-CoV-2 S protein and decreases the infectivity. The MH-1 peptide inhibits SARS-CoV-2 infection in mice by reducing viral load and suppressing immune responses in lung tissue, providing key experimental evidence for its antiviral activity. Although our study focuses on prophylactic intervention, future post-exposure treatment studies are needed to assess the therapeutic potential of MH-1. Furthermore, direct spike protein-HLA-C binding assays and validation against other SARS-CoV-2 variants are crucial for further strengthening the proposed mechanism and its clinical application.

## Supplementary Information

Supplementary Material 1.

## Data Availability

The datasets generated and analysed during the current study are available from the corresponding author on reasonable request.
