# Supplementary material for: HLA-C–derived peptide MH-1 as an early-stage intervention against SARS-CoV-2 infection
Source: Mol Med. 2026 Feb 16;32:48. doi: 10.1186/s10020-026-01434-3 (PMC13037026; doi:10.1186/s10020-026-01434-3)
Supplement: Supplementary file 1 — Supplementary Material 1. [file 10020_2026_1434_MOESM1_ESM.docx]

**Supplementary information**

1. **HLA-C knockdown**

Lentiviral particles with the lentiviral vector expressing HLA-C shRNA (#333639, #353148, and #363747) and scrambled shRNA were purchased from the RNA Technology Platform and Gene Manipulation Core (Academia Sinica, Taipei, Taiwan). Three target sequences for HLA-C shRNA lentiviral constructs are 5′-GCAACTTCTTACTTCCCTAAT-3′ (#333639), 5′-AGCTGTGGTCACCGCTATGAT-3′ (#353148), and 5′-GCAGAGATACACGTGCCATAT-3′ (#363747), respectively. The target sequence for scrambled shRNA sequence is 5′-CCTAAGGTTAAGTCGCCCTCG-3′. Jurkat cells were subcultured at 5 × 10^5^ cells/well into six-well tissue culture plates overnight. Cells were infected with lentiviral particles at a multiplicity of infection (MOI) of 5. To detect the interference effects of different target, HLA-C mRNA expression was determined using qPCR. Protein expression of HLA-C was evaluated by flow cytometry.

Knockdown efficiency of three HLA-C shRNA lentiviral constructs was evaluated at both the mRNA and protein levels. These three shRNA constructs reduced HLA-C mRNA expression by approximately 96.7% (#333639), 70.9% (#353148), and 72.8% (#363747), respectively. They also reduced HLA-C protein expression by approximately 93.9% (#333639), 55.1% (#353148), and 67.4% (#363747), respectively. Among them, shRNA #333639 demonstrated the highest knockdown efficiency and was selected for the subsequent viral infection.

**Table S1.** Mutations in the spike protein of the SARS-CoV-2-S Luc pseudotyped lentivirus**.**

| **Spike** | **WHO** | **Mutation(s) of Spike**† |
| --- | --- | --- |
| B.1.1.7 | Alpha | 69-70 del, Y144 del, N501Y, A570D, D614G, P681H, T716I, S982A, D1118H |
| BA.1.1.529 | Omicron | A67V, 69-70 del, T95I, G142D, 143-145 del, 211 del, L212I, ins214EPE, G339D, S371L, S373P, S375F, K417N, N440K, G446S, S477N, T478K, E484A, Q493R, G496S, Q498R, N501Y, Y505H, T547K, D614G, H655Y, N679K, P681H, N764K, D796Y, N856K, Q954H, N969K, L981F |
| BA.4/BA.5 | Omicron | T19I, L24S, 25-27 del, 69-70 del, G142D, V213G, G339D, S371F, S373P, S375F, T376A, D405N, R408S, K417N, N440K, L452R, S477N, T478K, E484A, F486V, Q498R, N501Y, Y505H, D614G, H655Y, N679K, P681H, N764K, D796Y, Q954H, N969K |
| XBB.1.16 | Omicron | T19I, L24S, 25-27 del, V83A, 144 del, G142D, H146Q, E180V, V213E, G252V, G339H, R346T, L368I, S371F, S373P, S375F, T376A, D405N, R408S, K417N, N440K, V445P, G446S, N460K, S477N, T478R, E484A, F486P, Q498R, N501Y, Y505H, D614G, H655Y, N679K, P681H, N764K, D796Y, Q954H, N969K |

†Information was obtained from the RNA Technology Platform and Gene Manipulation Core, Academia Sinica, Taipei, Taiwan

**Table S2**. A list of antibodies used in flow cytometry analysis.

| Antibodies (clone) | Source | Identifier | Dilution |
| --- | --- | --- | --- |
| ***Primary antibody (clone)*** |  |  |  |
| Goat anti-ACE-2 | R&D Systems | Cat# AF933,  RRID: AB_355722 | 1: 500 |
| Rabbit anti-TMPRSS2 (EPR24407-87) | Abcam | Cat# ab280567  RRID: AB_3720226 | 1: 200 |
| Mouse anti-HLA-C (DT-9) | Novus | Cat# NBP2-50419, RRID: AB_3326849 | 1:500 |
| ***Secondary antibody (For 1^st^ Ab detection)*** | |  |  |
| AF488 Rabbit anti-Goat IgG  (For ACE2 detection) | Thermo scientific | Cat# A-21222  RRID: AB_2535802 | 1:1000 |
| FITC Donkey anti-Rabbit IgG  (For TMPRSS2 detection) | Jackson ImmunoResearch | Cat# 711-095-152  RRID: AB_2315776 | 1:1000 |
| FITC sheep anti-mouse IgG  (For HLA-C detection) | Jackson ImmunoResearch | Cat# 515-095-062  RRID: AB_2340308 | 1:1000 |
| ***Control antibody*** |  |  |  |
| Goat IgG, polyclonal - Isotype Control | Abcam | Cat# ab37373 | 1:1000 |
| FITC Rabbit IgG - Isotype Control | Abcam | Cat# ab37406 | 1:1000 |
| FITC Mouse IgG1, κ Isotype Ctrl (FC) Antibody | Biolegend | Cat# 400110 | 1:1000 |

**Table S3.** Primer sequences used for quantitative-PCR.

| **Target gene** | **Primers** | **Sequences (5’-3’)** |
| --- | --- | --- |
| ***For viral load quantification*** | | |
| GAPDH | Forward  Reverse | 5’-CCCATGTTCGTCATGGGTGT-3’  5’-GGTCATGAGTCCTTCCACGATA-3’ |
| N gene of SARS-CoV-2 | Forward  Reverse | 5’-CACATTGGCACCCGCAATC-3’  5’-GAGGAACGAGAAGAGGCTTG-3’ |
| ***For HLA-C expression evaluation*** | | |
| GAPDH | Forward  Reverse | 5’-TTGCCCTCAACGACCACTTT-3  5’-TGGTCCAGGGGTCTTACTCC-3’ |
| HLA-C | Forward  Reverse | 5’-GGGTGGAGGCAGGTAAGAAAA-3’  5’-ATTCAGGATCGCAGACCAGC-3’ |


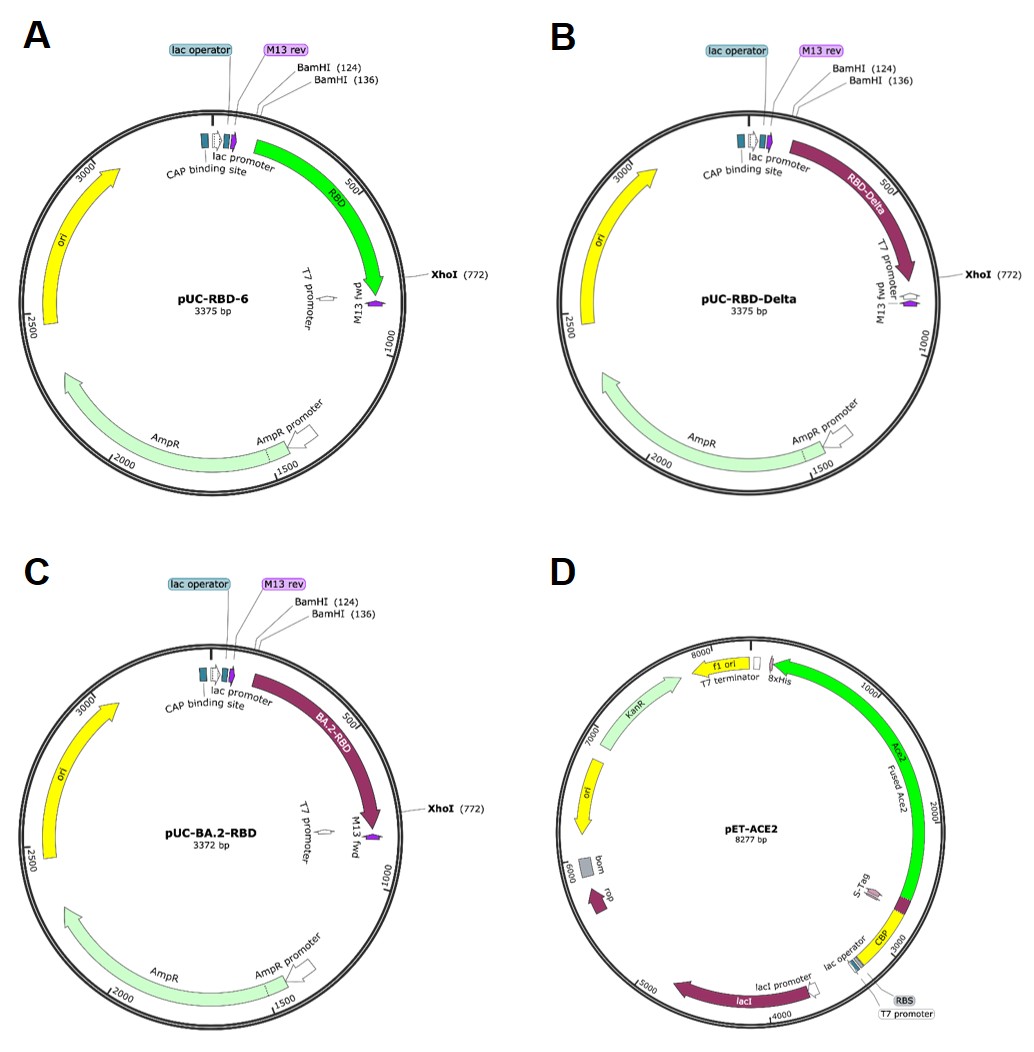


**Figure S1.** Construction of pUC expression vector maps for the S-protein receptor-binding domain (S-RBD) of SARS-CoV-2, such as (**A**) Wuhan, (**B**) Delta, (**C**) Omicron variants, and (**D**) ACE2.


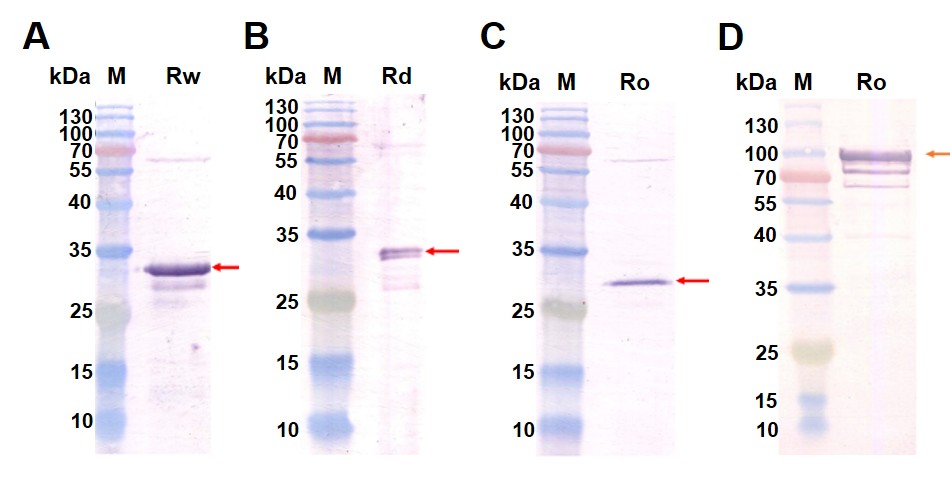


**Figure S2. Western blot images of the refolded SARS-CoV-2 S-protein receptor-binding domain (S-RBD, red arrow, molecular weight 26.72 kDa) of SARS-CoV-2.** (**A**) Wuhan strain, (**B**) Delta variant, (**C**) Omicron variant, (**D**) ACE2 (molecular weight 120 kDa). Protein bands were visualized using the NBT/BCIP chromogenic substrate (Sigma‑Aldrich, Cat. No. B1911).


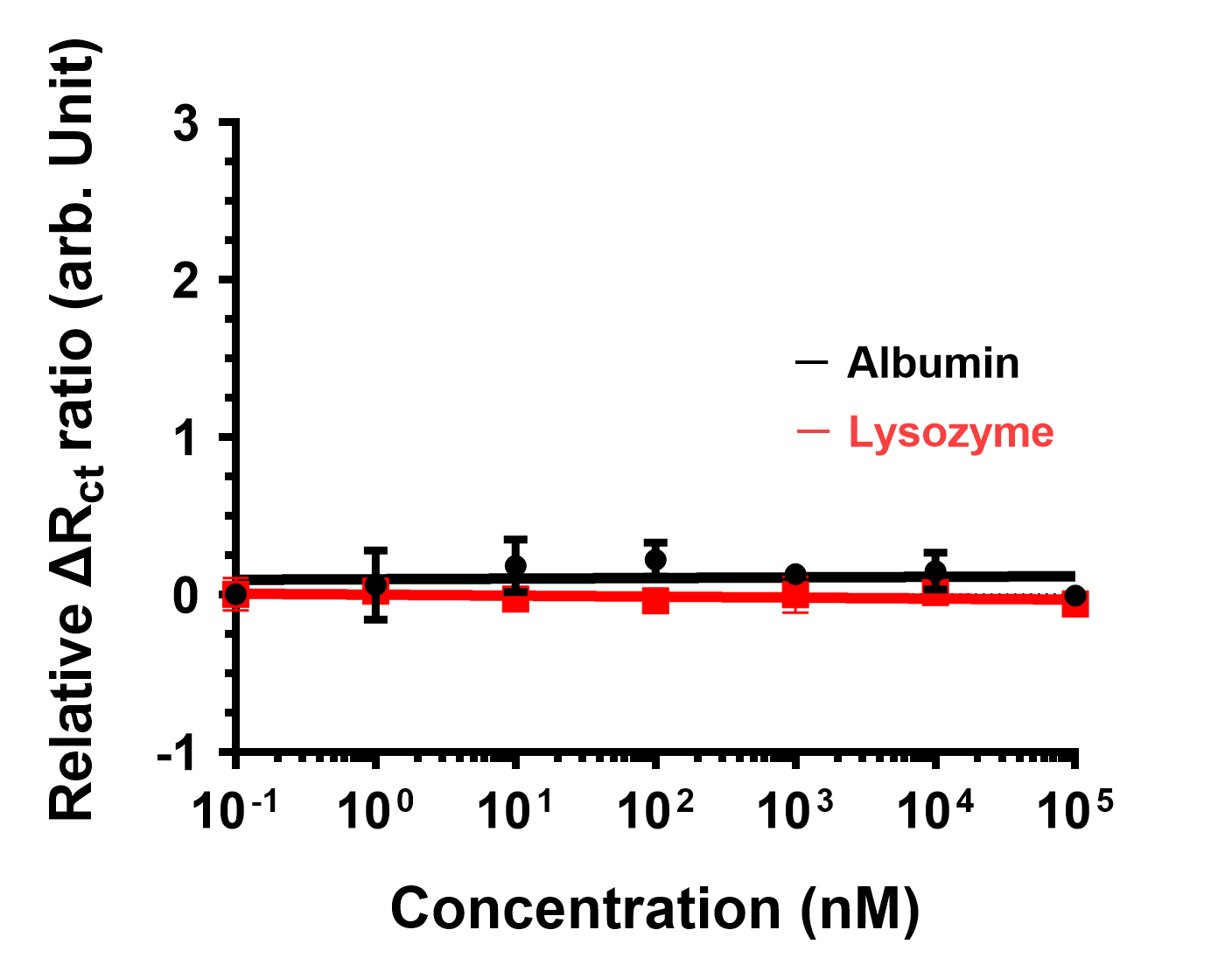


**Figure S3. Ligand-binding selectivity of the ACE2-Pd-NTFE-EIS biosensor.** The biosensor exhibited a selective interaction with the S protein, as evidenced by the concentration-dependent increase in ∆R_ct_, whereas no significant response was observed with albumin or lysozyme.


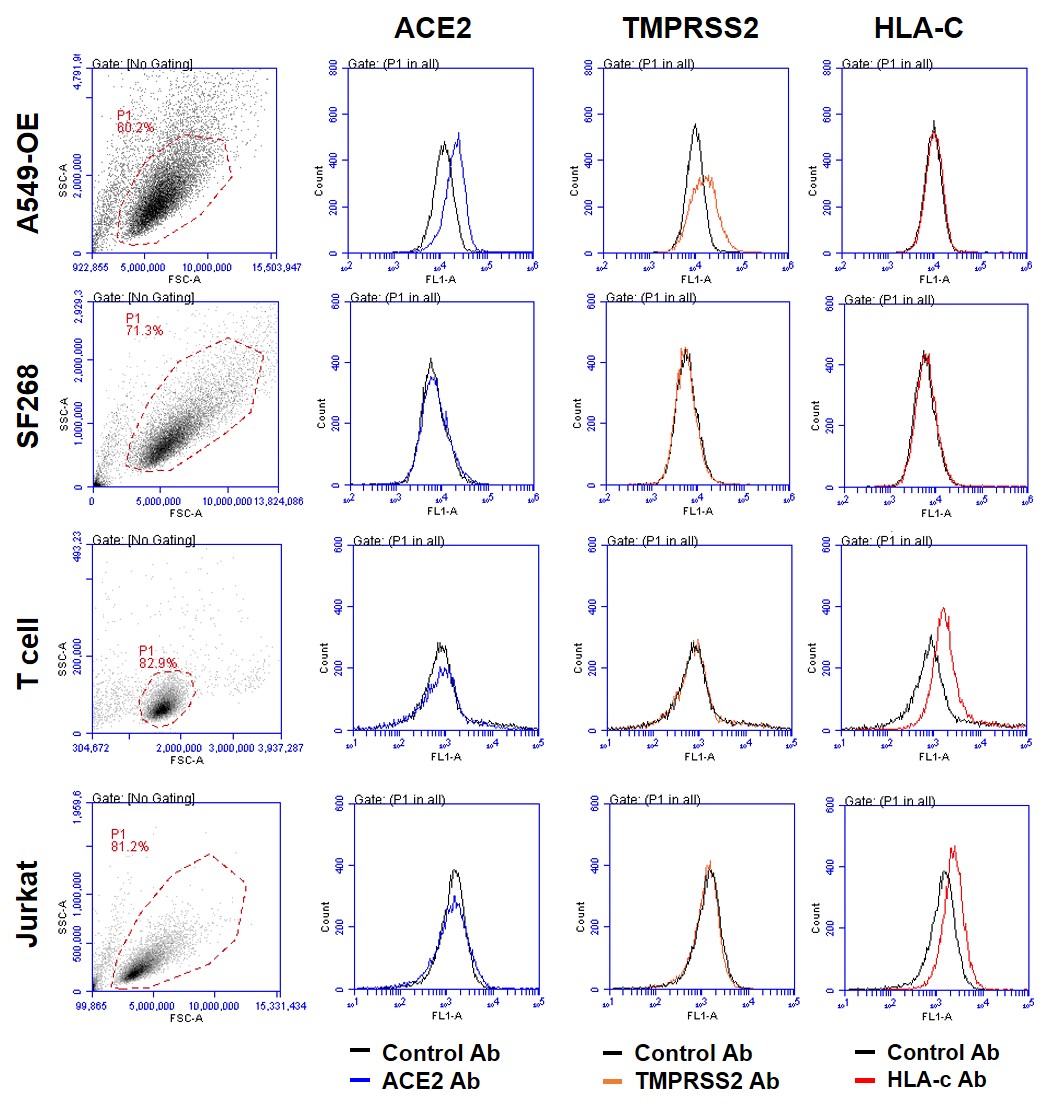


**Figure S4. Cell surface expression of ACE2, TMPRSS2, and HLA-C in different cell types.** Flow cytometric analysis of cell surface expression of ACE2, TMPRSS2, and HLA-C in A549-OE, SF268, primary T, and Jurkat T cells. Representative forward scatter/side scatter (FSC/SSC) plots illustrate the gated P1 populations used for analysis. Histograms show fluorescence intensity (FL1-A) for cells stained with specific antibodies against ACE2 (blue), TMPRSS2 (orange), or HLA-C (red), compared with corresponding isotype control antibodies (black). A549-OE cells exhibit high surface expression of ACE2 and TMPRSS2, whereas SF268 cells and T-cell populations display minimal to undetectable ACE2 and TMPRSS2 expression. In contrast, HLA-C is highly expressed in T cells.


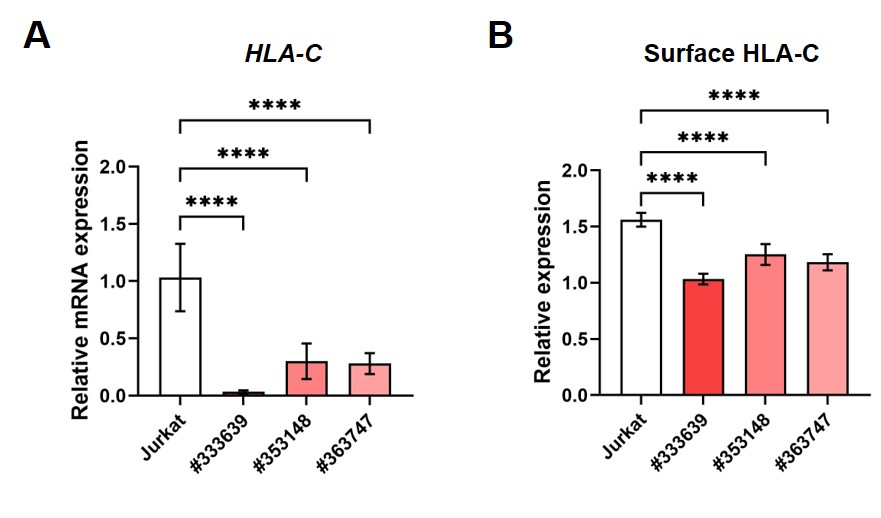


**Figure S5. The HLA-C knockdown efficiency of HLA-C shRNA lentiviruses in Jurkat cells.** (**A**) The mRNA expression of HLA-C was analyzed with q-PCR. Three HLA-C shRNA virus respectively treated in Jurkat cells, and collected to analyze HLA-C mRNA expression. (**B**) Flow cytometry analysis of HLA-C protein expression on Jurkat cells. ****: *p* < 0.0001, compared with the untreated (Jurkat) group.


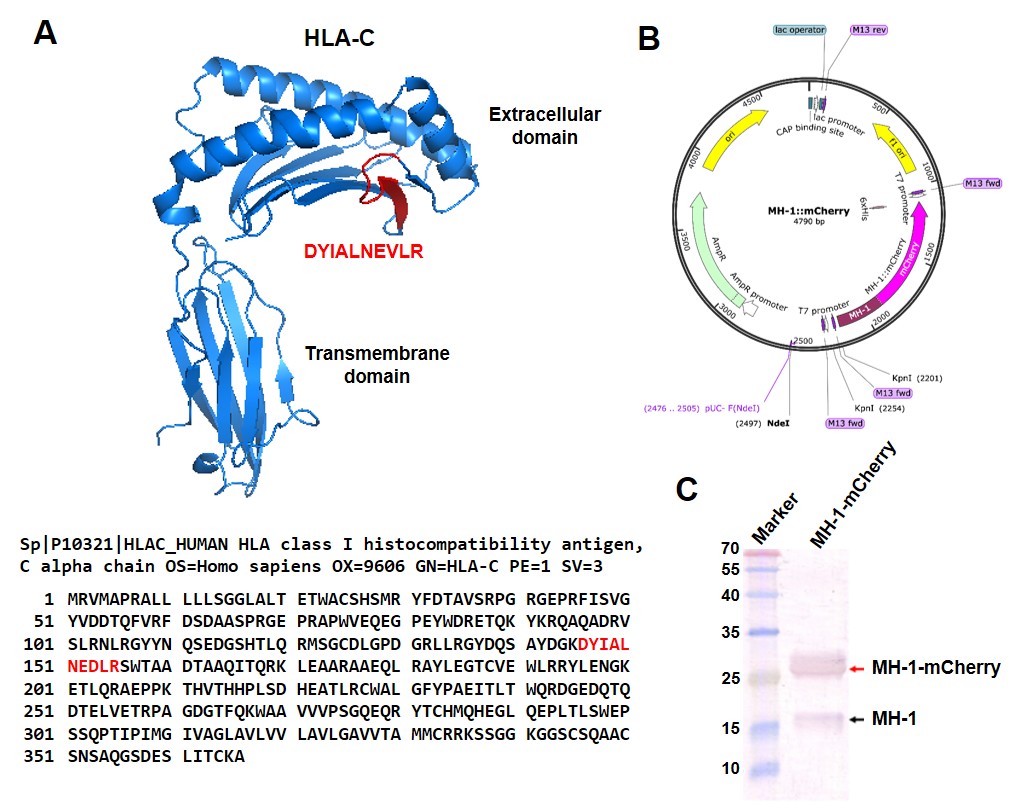


**Figure S6. Characterization, production, and identification of the MH-1 peptide.** (**A**) The peptide sequence of "DYIALNEVLR" is located in the extracellular domain of HLA-C. According to the HLA-C sequence, the HLA-C structure was modeled by the SWISS-MODEL web tool (<https://swissmodel.expasy.org/>). (**B**) The construction of the pUC expression vector for the MH-1 peptide. (**C**) Western blot images of the refolded MH-1 peptide. Black arrow: MH-1; Red arrow: mCherry. Protein bands were visualized using the NBT/BCIP chromogenic substrate (Sigma‑Aldrich, Cat. No. B1911).


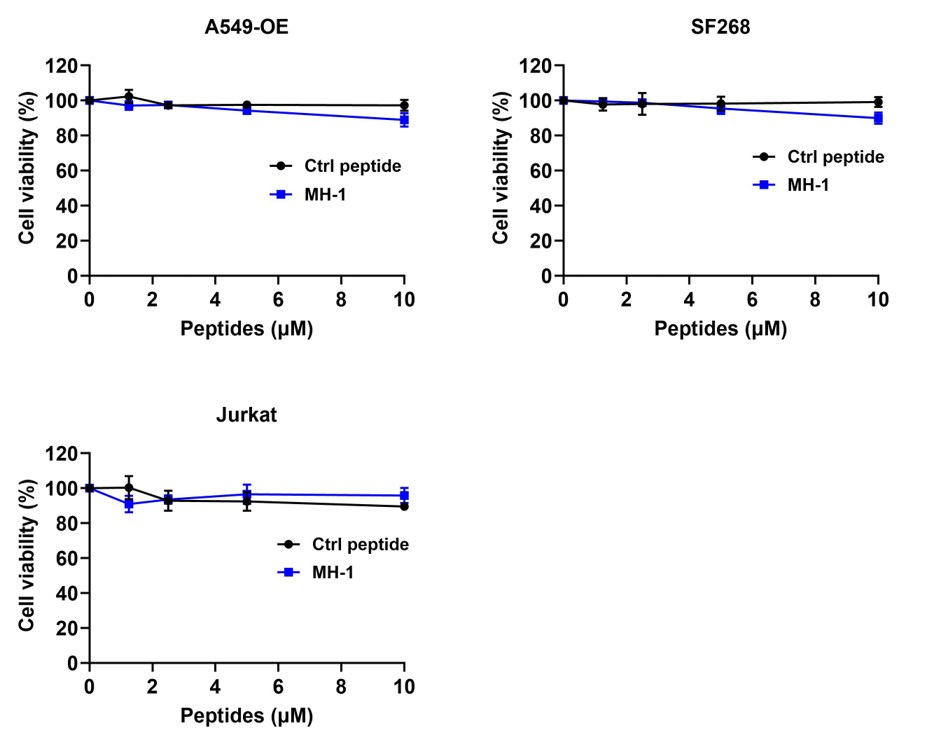


**Figure S7. The cytotoxicity of MH-1 at the specified test dose.** A549-OE, SF268, and Jurkat cells were treated with various concentrations of control peptide or MH-1 for 48 h. Their cytotoxicity was analyzed by MTT assay. The data are presented as mean ± standard deviation (SD) from three independent experiments.


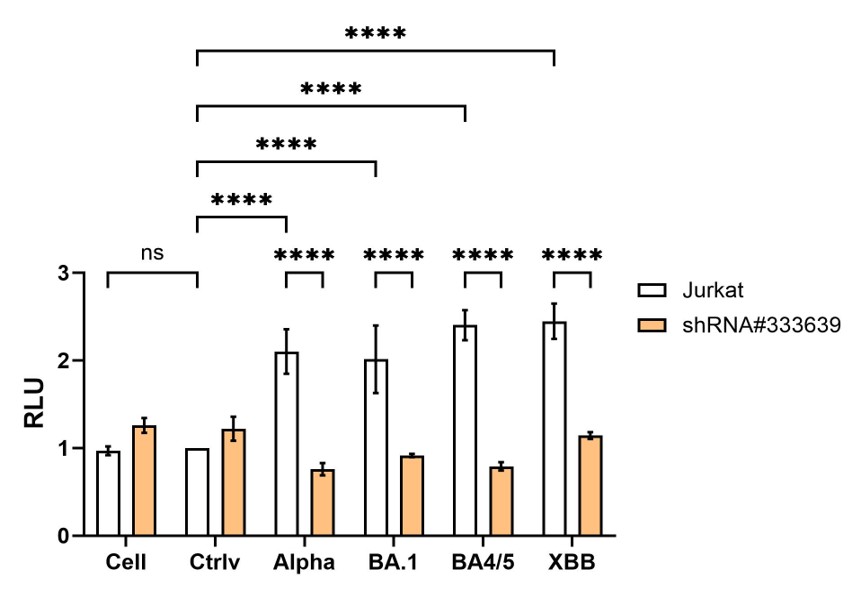


**Figure S8. Silence HLA-C expression by shRNA virus disrupted SARS-CoV-2 spike pseudotyped lentivirus infection.** Jurkat cells were pretreated with an ACE2 neutralizing antibody and a TMPRSS2 inhibitor for 1 h, then infected with SARS-CoV-2 spike pseudotyped lentiviruses, including Alpha (B.1.1.7), Omicron (BA.1), Omicron (BA.5), and Omicron (XBB.1.16) or control virus (Ctrlv, VSV-G) at MOI 0.5. Samples were collected 48 h post-infection. The data are presented as mean ± standard deviation (SD) from at least three independent experiments.


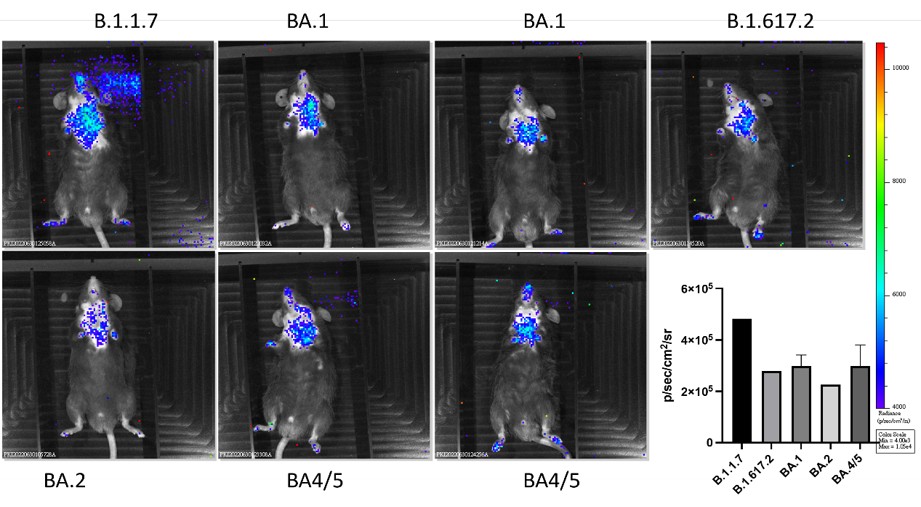


**Figure S9. Infectivity of the spike pseudoviruses in hACE2 transgenic mice.** Evaluation of the infectivity of various SARS-CoV-2-S Luc pseudoviruses, such as B.1.1.7, B.1.617.2, BA.1, BA.2, and BA.4/5. The IVIS imaging system detected the levels of virus present in the lungs.


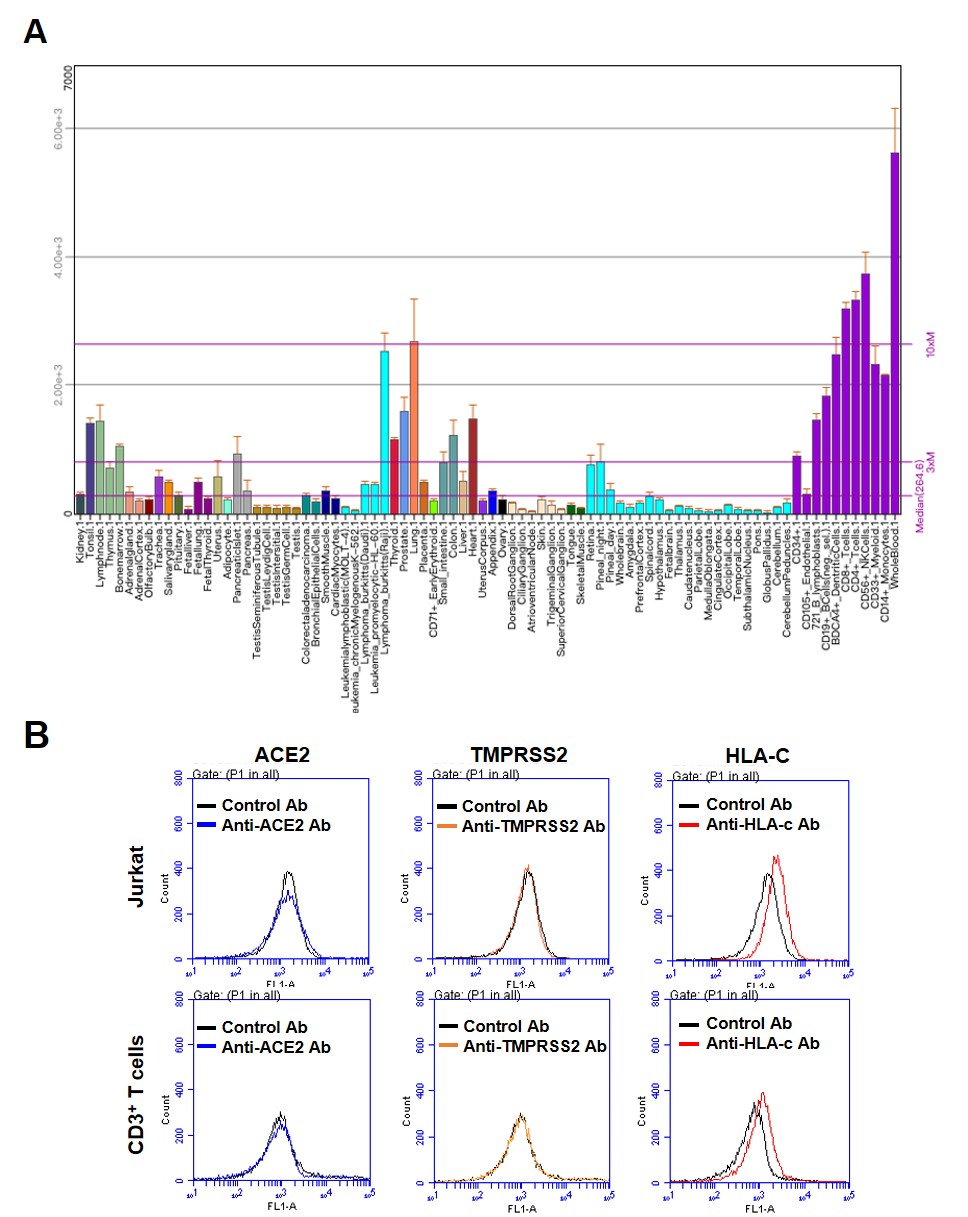


**Figure S10. HLA-C expression in T cells**. High levels of HLA-C were expressed in the immune cells (B-cells, T-cells, dendritic cells, NK cells, myeloid cells, and monocytes). Data were obtained from the Human Protein Atlas ([www.proteinatlas.org](http://www.proteinatlas.org/)).
